# The Stimulatory Gα_s_ Protein Is Involved in Olfactory Signal Transduction in *Drosophila*

**DOI:** 10.1371/journal.pone.0018605

**Published:** 2011-04-07

**Authors:** Ying Deng, Weiyi Zhang, Katja Farhat, Sonja Oberland, Günter Gisselmann, Eva M. Neuhaus

**Affiliations:** 1 Sino-France Joint Center for Drug Research and Screening, College of Life Science and Technology, Huazhong University of Science and Technology, Wuhan, P. R. China; 2 Cell Physiology, Ruhr-University Bochum, Bochum, Germany; 3 Bioduro (Beijing) Co. Ltd, Zhongguancun Life Science Park, Changping, Beijing, China; 4 Department of Cardiovascular Physiology, Georg-August University, Göttingen, Germany; 5 NeuroScience Research Center, Charité, Berlin, Germany; Freie Universitaet Berlin, Germany

## Abstract

Seven-transmembrane receptors typically mediate olfactory signal transduction by coupling to G-proteins. Although insect odorant receptors have seven transmembrane domains like G-protein coupled receptors, they have an inverted membrane topology, constituting a key difference between the olfactory systems of insects and other animals. While heteromeric insect ORs form ligand-activated non-selective cation channels in recombinant expression systems, the evidence for an involvement of cyclic nucleotides and G-proteins in odor reception is inconsistent. We addressed this question *in vivo* by analyzing the role of G-proteins in olfactory signaling using electrophysiological recordings. We found that Gα_s_ plays a crucial role for odorant induced signal transduction in OR83b expressing olfactory sensory neurons, but not in neurons expressing CO_2_ responsive proteins GR21a/GR63a. Moreover, signaling of *Drosophila* ORs involved Gα_s_ also in a heterologous expression system. In agreement with these observations was the finding that elevated levels of cAMP result in increased firing rates, demonstrating the existence of a cAMP dependent excitatory signaling pathway in the sensory neurons. Together, we provide evidence that Gα_s_ plays a role in the OR mediated signaling cascade in *Drosophila.*

## Introduction

Mammalian odorant receptors (OR) comprise the largest family of seven-transmembrane spanning G-protein-coupled receptors which are characterized by extracellular N- and intracellular C-termini [1]. OR proteins are localized in the cilia of the olfactory sensory neurons where they stimulate a Gα_olf_/cAMP-mediated signal transduction cascade ultimately leading to neuronal depolarization [2], [3]. 62 OR genes were shown to be expressed in the antennae, maxillary palps and larvae in *Drosophila melanogaster* [4]. Flies have therefore considerably fewer OR genes than vertebrates, most of which express between 600–1300 ORs [5]. The odor response spectrum for most receptors was determined *in vivo* by electrophysiological recordings [6]. The organization of the peripheral olfactory system shows striking similarities to the mammalian olfactory system, the ORs recognize multiple odors, the neurons express, with a few exceptions, one OR, and the axons of the olfactory neurons that express the same OR converge onto specific glomeruli in both, the insect antennal lobe and the mammalian olfactory bulb.

Nevertheless, differences exist in the functional properties of vertebrate and invertebrate OR proteins. One key difference is the ubiquitously expressed insect receptor OR83b, which is conserved across insect species [7]–[9]. OR83b interacts with conventional ORs and is essential to transport them to the sensory cilia [10]–[12]. Moreover, although *Drosophila* ORs were identified by bioinformatic strategies to contain seven transmembrane domains, recent experimental investigations have revealed that the membrane topology of *Drosophila* ORs is distinct from conventional GPCRs, with the N-terminus of these receptors located in the cytoplasm [13]. Similarly, insect gustatory receptors also lack clear sequence similarity to G-protein-coupled receptors (GPCRs). The hypothesis that insect chemoreceptors could define a novel family of transmembrane proteins was further substantiated by the findings that heteromeric insect OR/OR83b complexes can form ligand-gated ion channels [14]–[16]. Contradictory results concern the nature of the underlying transduction mechanism. While rapid, solely ionotropic, and G-protein independent currents were described by Sato *et al*. [14], non-selective cation currents activated by means of an ionotropic and a metabotropic pathway, with OR83b being directly activated by intracellular cAMP or cGMP, have been described in a study by Wicher *et al.* [16].

The existence and nature of a signal transduction cascade downstream of insect ORs, especially in the sensory neurons, is therefore a still partially unresolved question. Since they had been categorized as GPCRs during their initial identification, G-protein signaling has been investigated in odor elicited activation of insect neurons. Gα_q_ was found to be expressed in insect antennae, and although it is not specifically enriched in moth cilia [17]–[19], immunoreactivity was found in chemosensory hairs of *Drosophila* and *Anopheles* antennae [19], [20]. Also other heterotrimeric G-protein subunits have been found in the *Drosophila* antennae [21]. Pharmacological studies in locust, cockroach and moth antennae revealed that G-proteins are involved in odor-evoked increases in inositol 1,4,5-trisphosphate [17], [22], [23]. Reduction of Gα_q_ levels in *Drosophila* olfactory neurons by RNAi leads to impaired performance in several odor induced behavioural assays [24] and flies expressing mutated Gα_q_ showed reduced odorant evoked response in electrophysiological recordings [25]. Gαo was found to be required for maximal physiological responses to multiple odorants [26]. Also in gustatory neurons expressing receptors which also lack clear sequence similarity to GPCRs, experimental evidence exists for an at least modulatory role of heterotrimeric G-proteins in sugar perception [27]–[29]. On the other side, the results of a recent RNAi based study do not support a role for Gα proteins in odor sensitivity, although small influences of G-proteins on the odor evoked spike rates were observed for some odorants [30]. Genetic mosaic analysis used in this study showed that odor responses are normal in the absence of Gα_q_, which is required for normal CO_2_ responses [30].

We also aimed at determining if *Drosophila* ORs couple to G-proteins in the heterologous expression system and if G-proteins play a role in olfactory signaling *in vivo.* We found that the *Drosophila* Gα_s_ protein, a close homologue to the vertebrate olfactory G-protein Gα_olf_, plays a role in odorant induced signal transduction in the olfactory sensory neurons and characterized the role of the second messenger cAMP in insect olfactory neurons.

## Results

### Modulation of G-protein expression in the antennae

**Figure 1:**
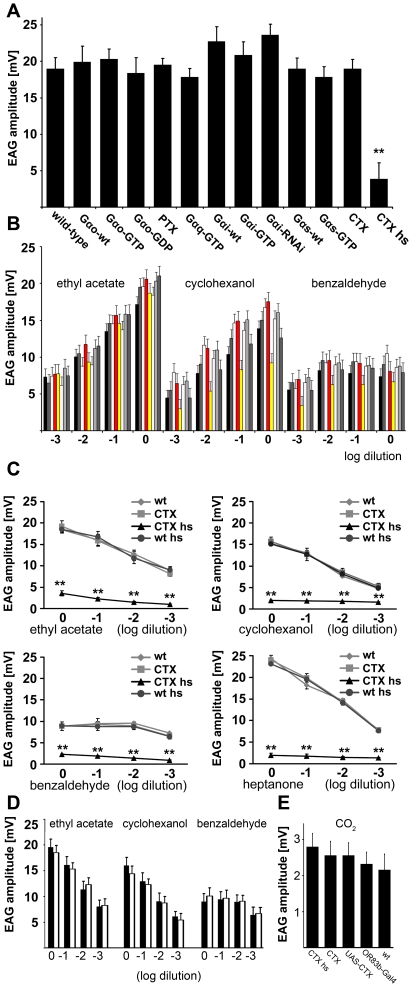
Screening G-proteins for participation in olfactory signal transduction. (**A**) UAS constructs of different Gα-proteins, mutated Gα-proteins and G-protein affecting toxins were expressed in the sensory neurons of the third antennal segment using an *Or83b-Gal4* driver line, last bars present heat shocked *Or83b-Gal4;UAS-CTX* flies (CTX hs) and *Or83b-Gal4;UAS-CTX* flies without heat shock (CTX). EAG amplitudes in response to application of undiluted ethyl acetate were recorded (n>10 flies were recorded). The difference between wt and heat shocked CTX flies was statistically checked by the unpaired Student's *t* test, **P≤0.01. (**B**) EAG amplitudes of different G-protein mutant flies (n>10 flies were recorded), expression of UAS constructs were driven by *Or83b-Gal4*. Odorant used were ethyl actetate, cyclohexanol and benzaldehyde, each in concentrations of 10^−3^, 10^−2^, 10^−1^, and undiluted. The fly strains tested were wt, PTX, Gαs wt, Gαs-GTP (marked in red), Gαq-GTP (marked in yellow), Gαo-GTP, Gαo-GDP, Gαo-wt (from left to right for each odorant dilution). If several doses were tested on the same antenna, lower doses were presented first, inter-stimulus intervals were at least 30 s. Significant differences were only observed for the responses of Gαq-GTP expressing flies towards cyclohexanol and low concentrations of benzaldehyde (shown in more detail in Figure S2A). Flies were statistically checked (pairwise) by unpaired Student's *t* tests; significance levels were set according to the Bonferroni post hoc test for *k* = 4 means,*P≤0.0125; **P≤0.0025. (**C**) EAG amplitudes (in mV) upon exposure to different concentrations of 4 odorants in wt flies, *Or83b-Gal4;UAS-CTX* flies (CTX), heat shocked *Or83b-Gal4;UAS-CTX* flies (CTX hs), and heat shocked wt flies (wt hs). The differences between CTX hs flies and wt, wt hs, CTX flies were statistically checked (pairwise) by unpaired Student's *t* tests; significance levels were set according to the Bonferroni post hoc test for *k* = 4 means, **P≤0.0025. (**D**) EAG amplitudes of heat shocked *UAS-CTX* flies without *Or83b-Gal4* driver (white bars) compared to non heat-shocked flies (black bars). (**E**) EAG amplitudes of heat shocked *Or83b-Gal4;UAS-CTX* flies (CTX hs, p = 0.04), *Or83b-Gal4;UAS-CTX* (CTX, p = 0.02), *UAS-CTX* (p = 0.26), *Or83b-Gal4* (p = 0.29), and wt flies to application of CO_2_ (according to significance levels for *k* = 5 means, * would have been assigned for P≤0.01). Error bars represent s.e.m.

An ongoing debate concerns the nature of the signal transduction cascade downstream of insect ORs. Since they had been categorized as GPCRs some time ago, G-protein signaling has been investigated in odor elicited activation of insect neurons, but conclusive genetic and biochemical evidence whether insect ORs couple to G-proteins is missing. We now aimed at determining if G-protein signaling is involved in odorant detection *in vivo.* In the *Drosophila* genome, several genes have been described that encode for Gα proteins: Gs, Gi, Gq, Go, Gf, and concertina *(cta),* which can all be detected in the antennae by RT-PCR (Figure S1 and [21]). We started by asking if, and if so which, G-protein(s) are involved in the olfactory signal transduction cascade. We expressed different mutated Gα proteins in the olfactory neurons by crossing UAS fly lines of different G-proteins and toxins interfering with G-protein function with an *Or83b* driver line. Screening of the F1 generation for olfactory deficits was performed using electroantennogram (EAG) recordings. Application of ethyl acetate elicited signals of normal amplitudes in flies overexpressing: wild type (wt) Gα_o_ (Gα_o_-wt), GTPase deficient (active) Gα_o_ (Gα_o_-GTP), constitutively GDP bound (inactive) Gα_o_ (Gα_o_-GDP), pertussis toxin (PTX), which inhibits specifically Goα in *Drosophila* by catalyzing ADP-ribosylation of the G-protein α-subunit [31], [32], GTPase deficient (active) Gα_q_ (Gα_q_-GTP), wt Gα_i_ (Gα_o_-wt), GTPase deficient (active) Gα_i_ (Gα_i_-GTP), wt Gα_s_ (Gα_s_-wt), and GTPase deficient (active) Gα_s_ (Gα_s_-GTP); summarized in Table S1. The only flies showing a severe defect in odorant induced signaling were those expressing cholera toxin CTX, an ADP-ribosyltransferase that activates Gα_s_-proteins [33] (Figure 1A). EAG amplitudes were partially reduced shortly (1 h) after activating CTX expression by heat shock (data not shown), and strongly diminished to up to 15% of the wt response after one day (Figure 1A). Also the detection of other odorants (cyclohexanol, benzaldehyde) was not altered in most tested mutant flies (Figure 1B), except for some odorant and concentration specific changes in flies expressing GTPase deficient Gα_q_ (Figure 1B and Figure S2A). Flies expressing constitutively active Gα_q_ showed alterations in the response amplitudes to cyclohexanol and to low concentrations of benzaldehyde, but not to ethyl acetate and higher benzaldehyde concentration (Figure S2A); the effects were much smaller than the ones measured in CTX expressing flies. Mutations in the *Drosophila* Gα_q_ have been shown before to significantly reduce the EAG amplitude in responses to odorants [25].

The CTX expressing mutant flies showed impaired responses to all tested odorants and all tested concentrations (Figure 1C). Gα_s_ is expressed in the forming nervous system of *Drosophila* embryos, and absence or mutation of Gα_s_ is lethal during development [34], suggesting that Gα_s_-dependent modulation of cAMP levels can play a critical role in a wide variety of neuronal processes throughout development. To ensure that activation of Gα_s_ by the expression of CTX does not interfere with proper formation of the sensory neurons during development, we controlled CTX expression by a *w [+]* flip-out cassette (*UAS* >*w [+]>CTX).* This cassette was later removed using *hsp70-flp,* which can be activated by heat shock [35]. To control for effects that may be caused by the heat shock itself, we tested wt flies, *Or83b-Gal4;UAS-CTX* flies (CTX), heat shocked *Or83b-Gal4;UAS-CTX* flies (CTX hs), and heat shocked wt flies with four different odorants, and found that only heat shocked CTX expressing flies showed olfactory deficits (Figure 1C and Table S2). EAG recordings from heat-shocked *UAS-CTX* flies without the *Or83b-Gal4* driver furthermore revealed that the UAS cassette used was not leaky (Figure 1D). Moreover, injection of CTX in the third antennal segment caused similar olfactory deficits as observed upon expression of CTX in the genetically modified flies (Figure S2B).

CO_2_ is sensed by a population of olfactory sensory neurons in the antennae of *Drosophila* which co-express a pair of chemosensory receptors, GR21a and GR63a, but do not express the olfactory co-receptor OR83b [36], [37]. Expectedly, CO_2_ responses were not altered in heat-shocked *Or83b-Gal4;UAS-CTX* compared to control flies (Figure 1 E).

### Single unit electrophysiology of CTX expressing flies

**Figure 2:**
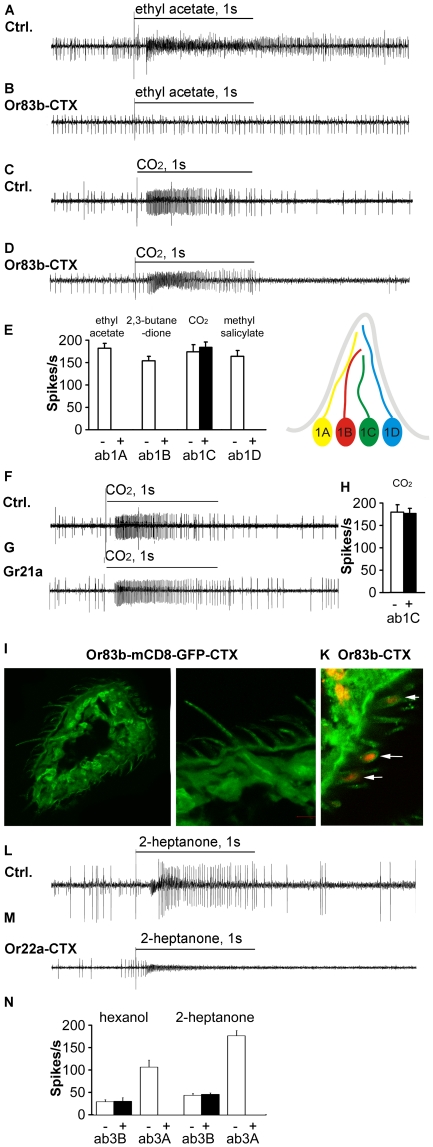
Gα_s_ mediates odorant signaling in fly olfactory neurons. Single unit recordings from the ab1 sensillum stimulated with ethyl acetate (diluted 1∶100) (**A, B**) and CO_2_ (**C, D**), known to activate two of the four neurons selectively. CO_2_ activates the ab1C neuron not expressing OR83b, ethyl acetate activates the ab1A neurons expressing OR83b. Ligand application in control flies (ctrl: *Or83b-Gal4;UAS-CTX* flies, no heat-shock) results in an increase in spike rates with both stimuli (**A, C**), whereas CTX expressing flies (*Or83b-Gal4;UAS-CTX* flies, heat-shocked) do respond only to CO_2_ (**B, D**). (**E**) Summary of single sensillum data for all four neuron types in the ab1 sensillum, odorants were diluted 1∶100, CO_2_ concentration was ∼14% (n>10 flies) and schematic representation of the ab1 sensillum bearing four neurons. White bars represent recordings from control flies (also labeled by - beneath the axis), black bars are responses from flies expressing CTX in *Or83b* neurons (also labeled with +). (**F**) CO_2_ response of the ab1C neuron of a control fly (*Or21a-Gal4;UAS-CTX* without heat shock) and (**G**) CO_2_ responses of the ab1C neuron expressing CTX (*Or83b-Gal4;UAS-CTX* flies, heat-shocked) remained normal after heat shock. (**H**) Summary of single sensillum data for ab1C neuron, CO_2_ was applied at ∼14% (n>10 flies). White bars represent recordings from control flies (also labeled by - beneath the axis), black bars are responses from flies expressing CTX in *Gr21a* positive neurons (also labeled with +). (**I**) Cellular morphology was normal heat shocked flies expressing CTX and mCD8-GFP under the control of the *Or83b* promoter. (**K**) Immunohistochemistry showed that OR83b (red, labeled with arrows) is correctly localized to the dendrite of flies expressing CTX under the control of the *Or83b* promoter. (**L**) Application of 2-heptanone to the ab3 sensillum activates both neurons (ab3A and ab3B) in control flies (*OR22a-Gal4;UAS-CTX* without heat shock). (**M**) Expression of CTX under the control of the OR22a promoter (*OR22a-Gal4;UAS-CTX* flies, heat-shocked) abolished the 2-heptanone response of the ab3A neuron. (**N**) Summary of single sensillum data for both neuron types in the ab3 sensillum, odorants (hexanol and 2-heptanone) were diluted 1∶100 (n>10 flies), white bars represent recordings from control flies (*OR22a-Gal4; UAS-CTX* without heat shock) (also labeled by - beneath the axis), black bars are responses from flies expressing CTX in Or22a positive neurons flies (heat shocked *OR22a-Gal4; UAS-CTX)* (also labeled with +). Error bars represent s.e.m.

EAG responses give only summated information of signal transduction events in olfactory neurons. In the next set of experiments we investigated how the activity of olfactory neurons was affected by CTX expression. We performed single-unit electrophysiology and measured the responses of olfactory neurons in the ab1 sensillum, which contains in total four neurons (ab1A-D) responding to different odorants (Figure S3). The ab1C neurons in the ab1 sensillum contains the chemosensory receptors GR21a and GR63a, but not the olfactory co-receptor OR83b [36], [37]. The ab1 sensillum therefore contains an internal control cell, since one of the neurons does not express the transgene when using the *OR83b-Gal4* driver line. Application of ethyl acetate, a known stimulus for the ab1A neuron, to the ab1 sensillum of control flies resulted in an increased spike rate, while the spike rates did not increase in ab1 sensilla of CTX expressing flies. The control spike trains shown in the figure come from non heat shocked *Or83b-Gal4;UAS-CTX* flies, which were indistinguishable from wt flies. Moreover, the ab1A, ab1B and ab1D neurons in CTX expressing flies showed no spontaneous spiking (Figure 2 B, E). As control, we determined the CO_2_ response of the same sensillum, since the ab1C neuron does not express OR83b or other ORs, but instead GR21a and GR63a together forming the CO_2_ receptor [36], [37]. Ab1C neurons showed no alteration in the spontaneous firing rates, and CO_2_ responses were found to be unaffected (Figure 2C, D), similar to the CO_2_ response in EAG recordings (Figure 1E). Odorants activating the remaining neurons, 2,3-butanedione for ab1B and methylsalicylate for ab1D showed the same effect as ethyl acetate in their inability to elicit increases in the spike rates in CTX expressing cells (Figure 2E).

To rule out that the effects are caused by general cellular toxicity of CTX, we expressed CTX specifically in the *Gr21a* expressing neuron in the ab1 sensillum. CTX expression did not change the response of the cells to application of CO_2_, ruling out that CTX expression causes cell poisoning or even cell death (Figure 2F–H). The absence of general cell poisoning of CTX was in agreement with another study investigating frizzled signaling [35]. Moreover, CTX expression did not disturb general cellular morphology in the antennae of *Gal4-Or83b; UAS-mCD8-GFP* flies (Figure 2I) or the presence of olfactory receptors in the dendrites of the sensory neurons (Figure 2K).

We also studied expression of CTX under the control of an OR specific Gal4 driver to target only a specific type of olfactory neuron. We used the *OR22a-Gal4* driver line to drive expression of CTX in the ab3A neuron of the ab3 sensillum, which contains in addition a second type of neuron (ab3B) expressing OR85b. The OR22a expressing ab3A neuron in control flies (*OR22a-Gal4;UAS-CTX*) responded to 2-heptanone and hexanol with an increased spike rate (Figure 2L, N), which was not observed in CTX expressing cells (in heat shocked *OR22a-Gal4;UAS-CTX* flies) (Figure 2M, N). The ab3B neuron in the same sensillum serves as positive control since it does not express OR22a (and is therefore not expressing CTX in heat shocked *OR22a-Gal4;UAS-CTX* flies) and responds to the same substances. Spiking rates in these cells were not changed in the heat-shocked mutants compared to mutant flies that were not heat shocked (Fig. 2N).

### GTPase defective Gα_s_ causes delayed desensitization of the odorant response

**Figure 3:**
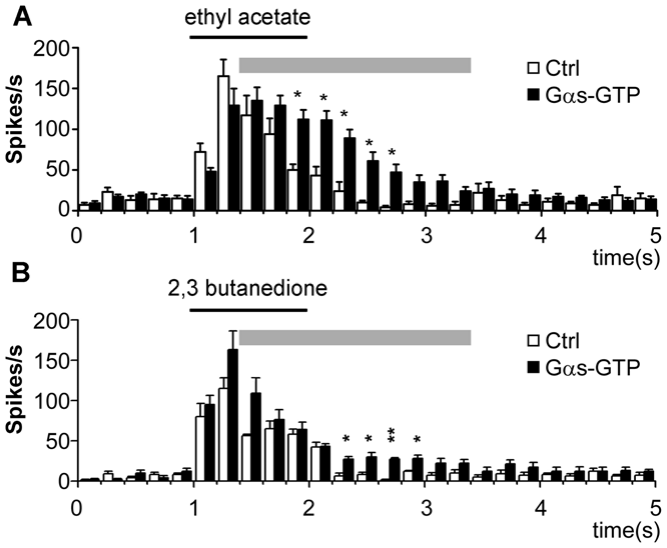
Expression of constitutively active Gα_s_. (**A**) Summary of the responses in single unit recordings of ab1A neurons in *Or83b-Gal4; UAS-Gα_s_-GTP* flies, expressing a GTPase deficient Gα_s_ mutant. Flies showed no difference in the initial increase in spike rates upon application of ethyl acetate (1∶100), but show a slower decay of the spike rates back to normal levels due to slowed GTP hydrolysis. The ligand was applied for 1s. (**B**) Similar results were obtained using 2,3-butanedione (1∶100) as ligand. Differences between the indicated data points (decay of the signal, grey bars) were statistically checked by the unpaired Student's *t* test, significance levels were set according to the Bonferroni post hoc test for *k* = 10 means (data points during signal decay, indicated by a grey line on top of the bar chart), **P≤0.005, *P≤0.001. Error bars represent s.e.m.

The experiments with CTX clearly point to a crucial role of Gα_s_ in olfactory signal transduction. In the following experiments we expressed mutated Gα_s_ subunits in olfactory neurons to further investigate this point. Since complete absence of Gα_s_ causes embryonic lethality in knock-out flies due to arrested development [34], investigation of olfactory perception in knock-out flies was not possible. We therefore tested *Or83b-Gal4; UAS-Gα_s_-GTP* expressing flies in single sensillum recordings. These flies express a GTPase defective Gα_s_ in the sensory neurons, in addition to the endogenous Gα_s._ The mutation leads to a prolonged activation of the G-protein and can be regarded as constitutively active. Recording the spike rates upon application of ethyl acetate to the ab1 sensillum revealed that expression of GTPase defective Gα_s_ caused a prolonged duration of the spike activity compared to the control flies (Figure 3A). A similar effect was observed upon application of 2,3-butadienone (Figure 3B). The prolonged duration of the period of increased firing in the mutant flies can likely be attributed to the deficiency in GTPase activity of the activated G-protein subunit.

Since cAMP was shown to increase the stimulus-dependent sensillar potential amplitude and the spontaneous action potential frequency in trichoid sensilla of the hawkmoth *Manduca sext*a [38], we specifically analyzed whether the responses might differ at the beginning of the application. We therefore analyzed the first 1000 ms of the response in a post-stimulus histogramm (Figure S4), grouping the spikes in 50 ms intervals. Also on this expanded time scale we could not see a significant rise in the mutant compared to wt control flies.

### Recombinantly expressed *Drosophila* ORs activate heterotrimeric G-proteins

**Figure 4:**
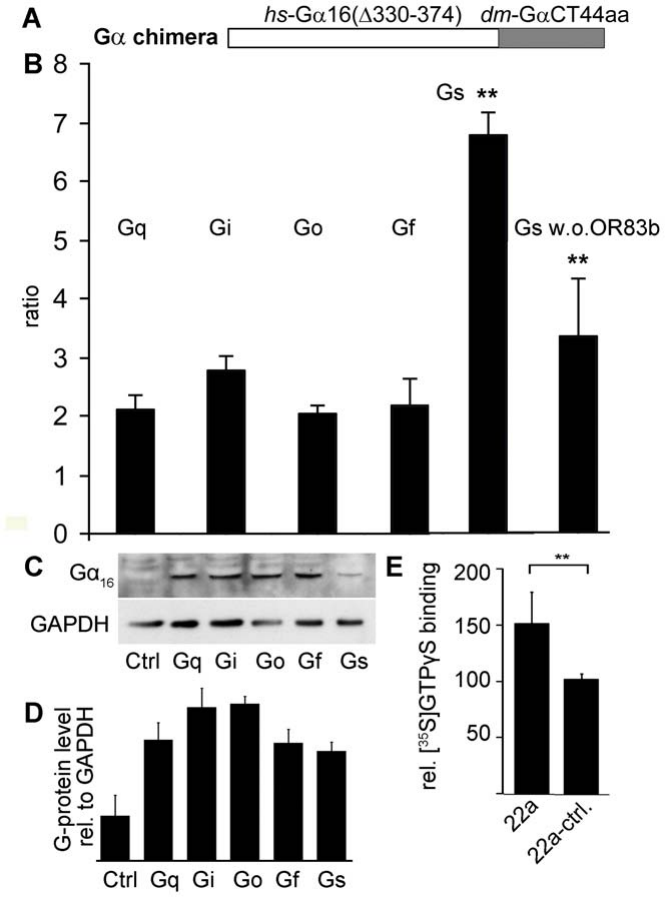
Role of G-proteins in signaling of recombinantly expressed olfactory receptors. (**A**) Chimeric construct with N-terminus of human Gα_16_ and the C-terminus of *Drosophila* Gα-proteins. (**B**) Ratio of transfected HEK293 cells responding to 500 µM cyclohexanone in Ca^2+^ imaging experiments; cells either express OR43a, OR83b and the respective G-protein chimera, OR43a alone and the respective G-protein chimera, or OR43a, OR83b and full length human Gα_16_. An increase in the ratio means that more cells responded upon co-expression of the G-protein chimera (n>5 independent transfections). The ratio determined with the Gα_s_ chimera was compared to the ratios obtained with the other G-protein chimera and was found to be signify-cantly different from all of them. (**C**) Western blot detection of recombinantly expressed G-protein chimera with an antibody against Gα_16_, equal amounts of protein was loaded per lane (controlled by GAPDH detection), control (ctrl) were non-transfected HEK293 cells. (**D**) Quantification of western blot analysis by densiometry, G-protein and GAPDH bands were ana-lyzed from cell preparations from 3 independent transfections. Band intensities originating from Gα_16_ stained membranes were divided by band intensities from GAPDH stained membranes. The small differences between the intensities from the different chimera were not significant (tested with Student's *t*-test). (**E**) [^35^S]GTPγS binding to membrane preparations upon odorant stimu-lation, cells were transfected with ORs and Gα-protein, control cells were mock transfected. The reaction was carried out in triplicates. Differences between the indicated data points were statistically checked by the unpaired Student's *t* test, **p<0.01. Error bars represent s.e.m.

For the understanding of the role of Gα_s_ in odorant signal transduction it is important to investigate whether *Drosophila* odorant receptors can activate heterotrimeric G-proteins in addition to their ion cannel function. To investigate the functional interaction between ORs and G-proteins, we tested whether co-expression of the *Drosophila* Gα-proteins could improve signaling in the recombinant HEK293 expression system, and whether this effect shows subunit specificity. To ensure efficient coupling of the *Drosophila* G-proteins to the endogenous Ca^2+^ pathway in HEK293 expression system, we constructed G-protein chimera composed of the N-terminus of human Gα_16_ lacking the last 44 amino acids, and the C-terminus of different *Drosophila* G-proteins (Figure 4A). The N-terminal Gα_16_ part of the protein contains the binding site for the effector proteins, all chimeric proteins should therefore couple to the same signaling cascade, whereas the C-terminal part should resemble the coupling specificity of different *Drosophila* Gα-proteins towards ORs. Similar constructs have already been described to enhance the signaling efficiency of vertebrate G-protein coupled receptors [39]–[41], and coexpression of similar chimeric G-proteins showed that the *Drosophila* sex peptide receptor couples to Gα_i_ and Gα_o_ in the recombinant expression system [42].

We coexpressed the respective chimeric G-proteins, OR83b and OR43a in HEK293 cells and performed Ca^2+^-imaging to record receptor elicited responses. The receptor under study was chosen, since it has been successfully used for Ca^2+^ imaging studies in recombinant expression systems previously [12], [43], [44]. We compared the number of cells responding to the application of cyclohexanone, a ligand for recombinantly expressed OR43a [44], to the amount of cells responding when the G-protein chimera were not coexpressed. For that we determined the ratio of cells responding with and without the chimeric G-protein. We coexpressed OR83b, OR43a and the respective G-protein chimera, or OR83b and OR43a without the G-protein determined the number of responding cells in each condition. The ratio of both data points revealed G-protein dependent differences. Although all chimeric G-proteins were able to moderately improve OR signaling, the Gα_s_ chimera had the strongest effect (Figure 4B). All G-protein chimeras were expressed at comparable levels in the recombinant expression system (Figure 4C, D). Non-transfected cells were used as control to show that no endogenous Gα-protein in HEK293 cells is detected under the same conditions. The presence of the G-protein chimera alone, with OR expression, did not result in any measurable Ca^2+^ increase upon odorant stimulation (data not shown). Next, we asked if the odor evoked Ca^2+^-signal and the enhancing effect by G-proteins was dependent on the presence of the OR83b subunit. For that we determined the ratio of cells responding with and without the chimeric Gα_s_ -protein, when only OR43a was expressed and again determined the number of responding cells in each condition. We found that expression of OR43a alone in HEK293 cells was sufficient to generate cyclohexanone-dependent cellular responses, which were greatly enhanced by Gα_s_ coexpression (Figure 4B). These findings provide additional indications that the receptor proteins indeed interact with G-proteins and that the odorant sensing subunit might be the site of interaction.

In another approach, we transfected HEK293 cells with odorant receptors (OR83b and OR22a), made membrane preparations and assayed [^35^S]GTPγS binding, which measures the level of G-protein activation following agonist occupation of a GPCR. We performed this assay on HEK293 cell membranes transfected with OR22a, OR83b and *Drosophila* Gα_s_. Membranes were stimulated by a mix of the OR22a ligands pentyl acetate and 1-octen-3-ol [6], which did not give any unspecific responses in this assay in HEK293 cells. Although these substances are not the best known ligands for OR22a [45], we identified them as best suited for the recombinant experiments conducted here. Incubation with the cognate ligands lead to a significant increase in membrane bound radioactivity in comparison to control membranes, indicating that ligand binding to OR22a is indeed activating the G-protein (Figure 4E). Similar results were obtained when OR43a was coexpressed with OR83b (data not shown). Although ligand induced GTPγS binding was weaker in OR43a expressing cells, there was no indication for receptor specific effects in these experiments.

### Gα_s_ is localized in the dendrites of the olfactory sensory neurons

**Figure 5:**
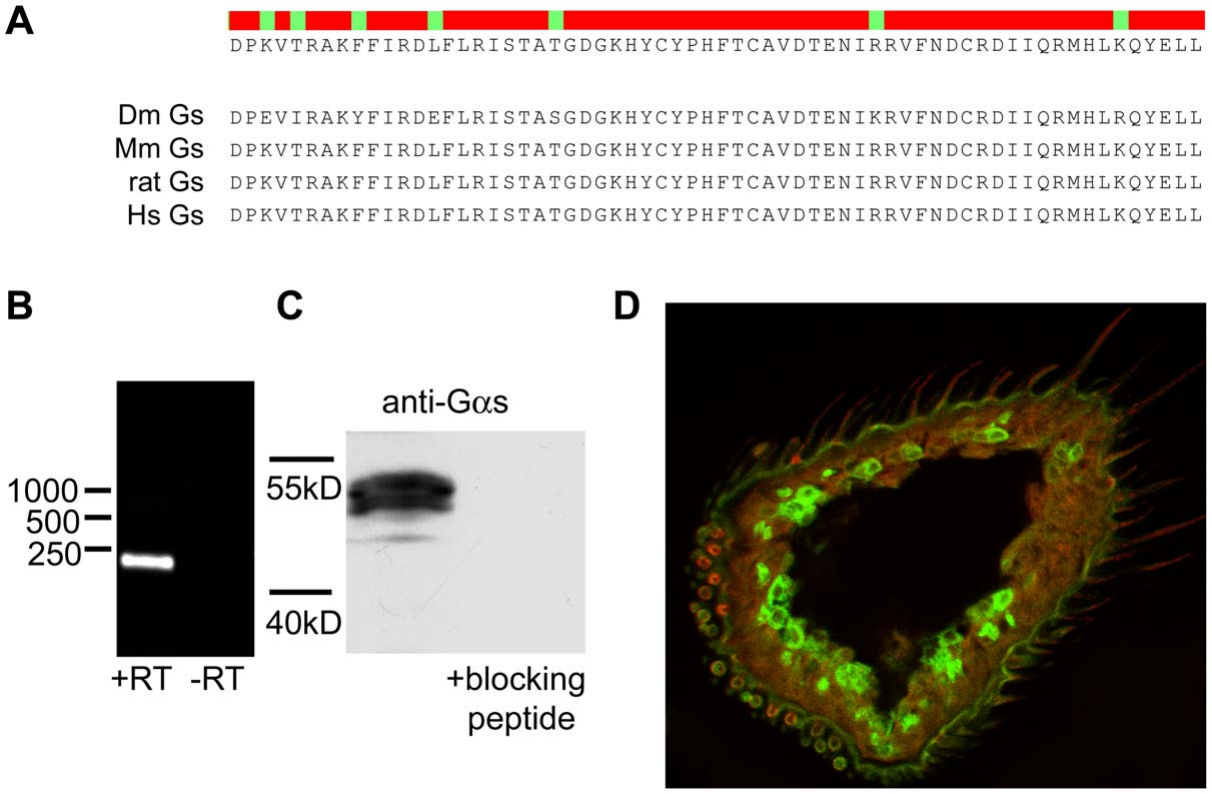
*Drosophila* Gα_s_ is localized in the sensilla of the olfactory sensory neurons. (**A**) Alignment of C-termini of Gαs from different species showing the high degree of conservation in this part of the protein. (**B**) RT-PCR with RNA prepared from manually collected antennae using Gα_s_ specific primers (+ RT), control lane is RNA without reverse transcription (-RT). (**C**) Western blot of manually collected antennae probed with anti-Gα_s_ showing a band approximately 50 kD, which disappears after pre-incubation of the antibody with a specific blocking peptide. (**D**) Staining of the third antennal segment using Gα_s_ specific antibodies. Red - Gα_s_; Green – mCD8-GFP (*Or83b-Gal4; UAS-mCD8-GFP*).

To understand the possible *in vivo* function of Gα_s_, it is necessary to find out whether it is expressed in olfactory neurons and where it is localized. This is a prerequisite for a direct activation by ORs and participation in the signal transduction cascade. We performed immunohistochemistry, RT-PCR, and western blotting to investigate the presence and localization of Gα_s_ in the *Drosophila* antennae (Figure 5). Gα_s_ mRNA could be detected in the third antennal segment by RT-PCR (Figure 5B). *Drosophila* Gα_s_ shows strong homology to vertebrate Gα_olf_ at the C-terminus (Figure 5A). It could therefore be detected in the third antennal segment by western blotting using an anti-vertebrate Gα_olf_ antibody (Figure 5C). In sections of *Gal4-Or83b; UAS-mCD8-GFP* flies we could localize Gα_s_ in the dendrites of OR83b expressing neurons (Figure 5D).

### cAMP causes increased spike rates in olfactory sensory neurons

**Figure 6:**
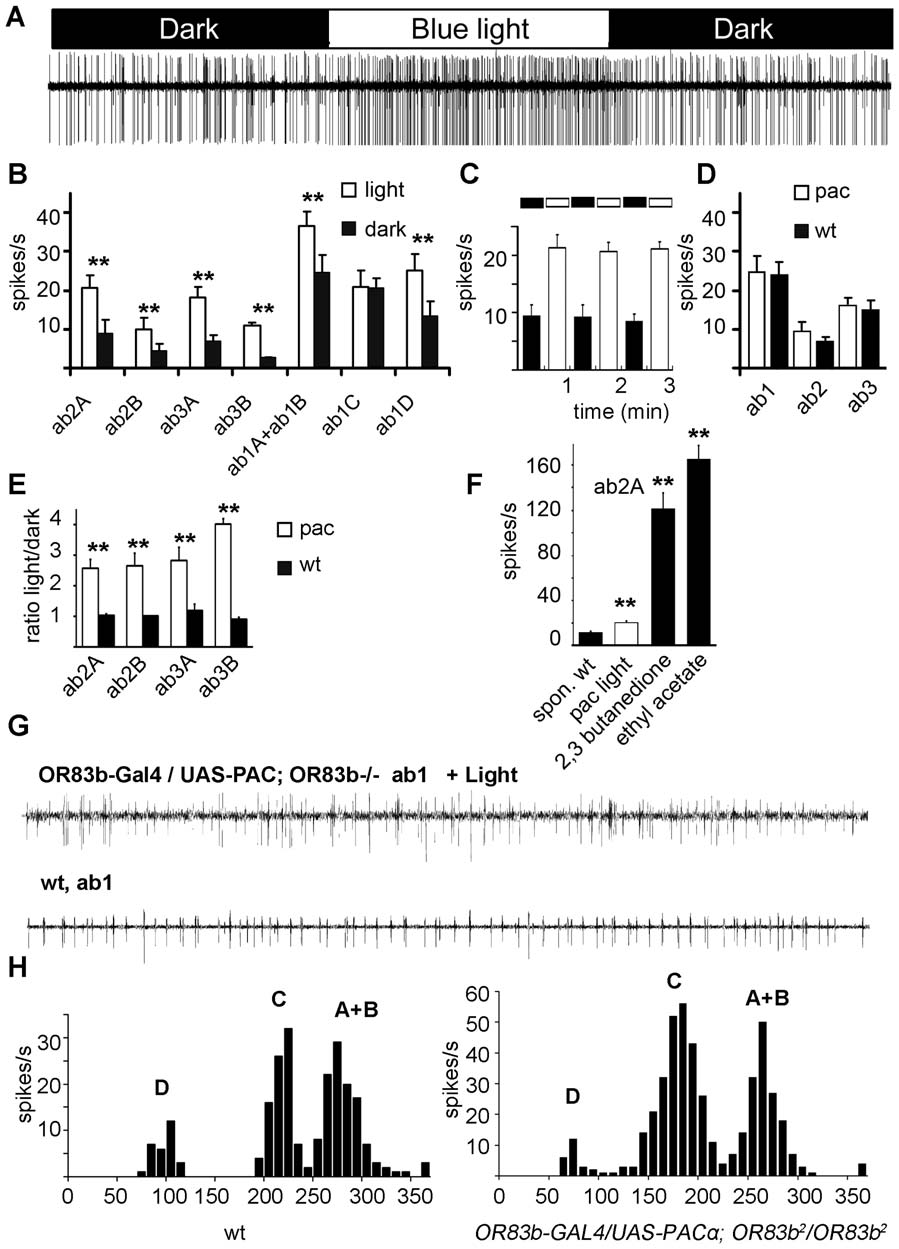
Increased cAMP levels can recover the spontaneous activity of olfactory neurons. (**A**) Increase in spike rates in single unit recordings from the ab2 sensillum in *Or83b-Gal4; UAS-Pacα* flies upon transition from dark to strong blue light excitation showing that cAMP increase stimulates an excitatory pathway in olfactory neurons, the increase was rapidly reversible. (**B**) Summaries of single unit recordings from the different neurons in the ab1, ab2 and ab3 sensilla. (**C**) Reversibility of the response to light shown by repeated 30 s on/off cycles in ab2A neurons. (**D**) Summaries of single unit recordings from ab1, ab2 and ab3 sensilla from wt (white bars) and *Or83b-Gal4; UAS-Pacα* flies (black bars) recorded in the dark. (**E**) Ratios of spike rates for ab2A, ab2B, ab3A and ab3B neurons recorded under blue light illumination vs. dark, showing the relative increases in spike rates in wt (black bars) and *Or83b-Gal4; UAS-Pacα* flies (white bars). (**F**) Comparison of the light induced spike rate in the ab2A neuron in *Or83b-Gal4; UAS-Pacα* flies (white bar) to spontaneous and odor induced spike rates in wt (black bars) flies. (**G**) Single unit recording of wt ab1 sensillum and of the blue light illuminated *Or83b-Gal4/UAS-PACα; Or83b^2^/Or83b^2^* ab1 sensillum. (**H**) Analysis of the of spike amplitude distribution recorded from ab1 sensilla in wt and *Or83b-Gal4/UAS-PACα; Or83b^2^/Or83b^2^* flies. Data shown represent about 450 spikes form 8 s recording. “A”–“D” indicate subpopulations of spikes attributed to neurons A, B, C and D. Differences between the indicated data points were statistically checked by the unpaired Student's *t* test, *p<0.05; **p<0.01. Error bars represent s.e.m.

Since Gα_s_ most likely affects cAMP mediated signaling in the antennae, we tested whether an increase in intracellular cAMP in the sensory neurons would also cause an increase in the spike rates. We expressed the photoactivated adenylyl cyclase (PAC) from the flagellate *Euglena gracilis* in the olfactory neurons, a protein recently reported to yield light-induced changes in cAMP levels in *Drosophila melanogaster* [46]. Since the activity of PAC is strongly and reversibly enhanced by blue light, we recorded spike rates from ab2 sensilla in the dark and exposed the antennae to blue light. The light exposure driven increase in cAMP resulted in an increase in the spiking rate of the neurons (Figure 6A). Light induced increases in spiking rates were detected in several types of sensilla (Figure 6B). The effect was reversible, and the return to normal spike rates occurred rapidly after turning the blue light off, this cycle could be repeated several times (Figure 6C). The activity of PAC in the olfactory neurons was very low without activation by blue light (Figure 6D). In ab2A, ab2B, ab3A and ab3B neurons, the light induced increase in spike rates was significantly higher than in wt flies, showing that the increase in spike rates upon blue light exposure depends on PAC expression (Figure 6E). PAC activation induced lower spike rates in ab2A neurons than the undiluted odorants ethyl acetate and 2,3-butanedione, which are strong activating odorants (Figure 6F). Since the excitation light has to pass the cuticle of the third antennal segment, and since the activation of PAC and generation of cAMP depends on the intensity of the light used [46], stronger neuronal excitation can be expected when light sources with higher intensities would be used. Cells activated by blue light could be further activated by odorant exposure (data not shown).

To test whether an increase in cAMP levels in the sensory neurons would lead to an increased spiking rate in OR83b knock-out flies (*Or83b* ^-/-^), we generated a mutant fly expressing PACα in the neurons on a OR83b knock-out background. Single sensillum recordings from the ab1 sensillum of *Or83b^-/-^* flies showed only one group of spikes representing the activity of the *Gr21*a neuron (Figure S5). The ab1 sensillum in wt flies showed four groups of spikes, corresponding to the four cell types projecting their dendrites in this sensillum (see Figure 2). When PACα was expressed by the *Or83b-Gal4* driver in *Or83b^-/-^* flies, and the fly antennae were exposed to blue light, additional neuronal activity could be recorded (Figure 6G). Neither PAC expression alone (without stimulation by blue light), nor light exposure to *Or83b^-/-^* flies resulted in significant changes in spiking activity of the neurons (Figure S5). Also CO_2_ responses in PAC expressing *Or83b^-/-^* flies were not significantly changed, and odorant responses were still absent (Figure S5). We next sorted all spikes in a given time interval and analyzed the spike amplitude distribution (Figure 6H). We observed a similar spike distribution pattern in 453 spikes analyzed from 8 seconds recording of the ab1 sensillum of an *Or83b-Gal4/UAS-PACα; Or83b^2^/Or83b^2^* (*Or83b^-/-^)* fly and 229 spikes from a wt fly (Figure 6H), indicating that PACα mediated cAMP rise in olfactory receptor neurons does efficiently recover spontaneous neuronal activity.

## Discussion

*Drosophila* ORs are seven transmembrane proteins which were originally classified as G-protein coupled receptors. The receptors have an unusual membrane topology with an intracellular N- and extracellular C-terminus [13], [47], and can function as ligand-gated ion channel in recombinant expression systems [14], [16], raising the question of whether these receptors also use G-proteins for signal transduction. In the present study, we investigated whether *Drosophila* ORs couple to G-proteins. Screening an array of different over-expressed G-proteins, mutated G-proteins, and G-protein affecting toxins resulted in the identification of Gα_s_, a close homologue to the vertebrate olfactory G-protein Gα_olf_, as being important for odorant induced signal transduction in the olfactory sensory neurons. Sustained expression of cholera toxin (CTX), which ribosylates the Gα_s_ subunit of the heterotrimeric G-protein and results in constitutive cAMP production, caused a reduction in electroantennogram (EAG) recordings and abolished firing rates of olfactory neurons *in vivo*. Moreover, expression of GTPase defective Gα_s_ caused a prolonged duration of spike activity when compared to wt flies. Recombinantly expressed ORs could directly activate G-proteins (measured by odorant induced [^35^S]GTPγS binding to membrane preparations). Moreover, co-expression of chimeric Gα_s_ with OR83b and OR43a in HEK293 cells lead to an increased cellular activation upon ligand exposure. Finally, the role of cAMP as a second messenger in the sensory neurons was further demonstrated by expression of photoactivated adenylate cyclase.

### G-protein signaling in *Drosophila* olfactory neurons

In the initial screening procedure, CTX expressing flies showed strong reduction of odorant induced signals in EAG recordings. Single sensillum recordings revealed an absence of spontaneous neuronal activities in CTX flies, which was not caused by general cell poisoning effect of the toxin. Interestingly, background spiking is also absent in *Or83b-/-* flies, again not caused by unspecific cellular damage but by deficient receptor transport, indicating that disturbance of single components of the signaling cascade can lead to profound effects on neuronal spiking. After single sensillum recordings of Gα_s_-GTP flies, which express a constitutively active mutated Gα_s_-protein with decreased GTPase activity, we observed differences in the time course of odor induced spike rates compared to wt flies. The prolonged firing of the neurons in the mutant flies was likely caused by prolonged G-protein activation due to the deficiency in its GTPase activity. Taken together, the electrophysiological recordings of the mutant flies showed that Gα_s_ is involved in odorant detection in *Drosophila* olfactory neurons.

Yao and Carlson agree to a large extend to our finding that odor evoked initial spike rates of ab1A, ab2A, and ab3A were not altered by expression of GTPase defective Gα_s_ [30]. In addition, we found that expression of GTPase defective Gα_s_ caused a prolonged increase of the spike rates compared to the control flies, which is likely caused by inefficient shut off of the response due to the deficiency in GTP hydrolysis. Similar to the study published by Yao and Carlson [30], we did not find altered odor evoked EAG amplitudes or spike rates in Gα_s_ RNAi expressing olfactory neurons (data not shown). In our view this can be explained by inefficient reduction of G-protein levels. This is consistent with our observation that flies expressing Gα_s_ RNAi under a pan-neuronal driver are viable (data not shown), while Gα_s_ knock-out flies are not [34]. Mosaic analysis with a repressible cell marker showed that Gα_s_-/- olfactory neurons had a decreased response to near-saturating concentrations of methyl butyrate and butyric acid, which can be rescued by Gα_s_ expression [30]. Although the contribution of Gα_s_ to odor responses was very modest in the study by Yao and Carlson [30], observable effects were reported.

Conflicting models for OR signaling have been suggested. Two studies found that OR and the ubiquitously co-expressed receptor OR83b form a ligand gated ion channel in heterologous expression systems [14], [16], however one of these groups proposed that ORs may still couple to G-proteins [48]. Data from another recent report do not support a direct role of G-protein signaling for the initial response of olfactory neurons [30], although Gα_o_ was found to be required for maximal responses to several odorants [26] and in particular Gα_q_ was earlier found to be required by ab2A neurons for full sensitivity to low concentrations of odors [25]. The latter finding is consistent with the odor concentration-dependent differences we found in the Gα_q_ mutant flies. There is moreover compelling evidence from many studies for an involvement of the Gα_q_ effector enzyme PLCβ in signal transduction cascades in pheromone and odor transduction in moths (reviewed in [49]). Precisely how the alternate mechanisms of odor transduction interact is unclear at the moment, but different odors and strengths of stimuli could in principle stimulate different transduction pathways to varying extent, and colocalized signal transduction cascades could allow adjustments in response to endogenous physiological rhythms, behavioral states, and stimulus properties [49]. *Drosophila* OR59b for example was shown to mediate excitatory and inhibitory responses in response to different odorants [6]. Different second messengers may therefore modulate the odor response depending on stimulus length and strength.

### G-protein in signaling of recombinantly expressed ORs

In addition to the physiological experiments showing the involvement of Gα_s_ in odorant detection, we demonstrated that signaling of ORs in the HEK293 system is enhanced upon co-expression of a *Drosophila* Gα_s_ chimera. This finding provides additional indications that the receptors may interact with G-proteins. *Drosophila* receptors might even use a similar coupling mode as other GPCRs, since the G-protein chimera where only the C-termini of human Gα_16_ was exchanged with the respective *Drosophila* G-proteins were sufficient to enhance the signaling properties of the ORs. The fact that we found increased binding of [^35^S]GTPγS when ORs and G-protein were co-expressed in HEK293 cells indicates that direct coupling may occur, since no other components *Drosophila* olfactory neurons were co-expressed.

Recent work on recombinantly expressed ORs showed that inhibitors of heterotrimeric G-proteins (GDP-β-S), adenylate cyclase, guanylyl cyclase, cyclic nucleotide phosphodiesterases and phospholipase C have negligible impact on OR43b responses, which lead the authors to the conclusion that G-protein signaling does not play a role in cell-based assays with insect ORs [15], [50]. Activation of mammalian ORs in HEK293 cells leads to a transient inositol phosphate production followed by an increase in intracellular Ca^2+^ that can be detected at the single-cell level using the Ca^2+^-sensitive dyes and ratiofluometric imaging techniques [51], [52]. Only when Gα_olf_ is cotransfected, vertebrate ORs can efficiently couple to a cAMP based signal cascade in recombinant expression systems [53]. Negligible effects of adenylate cyclase, guanylyl cyclase and cyclic nucleotide phosphodiesterases do therefore not exclude the possibility of G-protein coupling, neither does GDP-β-S incubation, since the drug is barely membrane permeable. Although the membrane topology of the *Drosophila* ORs is nicely demonstrated [13], [15], this experimental approach to signal transduction in the recombinant expression system does not unambiguously rule out a role for G-proteins but rather demonstrates that the insect receptors are ligand gated ion channels.

What has to be investigated in more detail is whether productive interaction with the G-protein only occurs when the receptors dimerize or whether monomers, and if so which part of the monomer, can activate the heterotrimeric G-protein. Our experiments in the HEK293 cell system suggest that the odorant sensing subunit of the receptor is sufficient for G-protein coupling, even though the effect of G-protein co-expression was stronger for heteromultimeric receptors. While ion channel characteristics depend on heteromeric complexes of a conventional insect ORs and the highly conserved OR83b family co-receptor [14], several reports support the activation of ORs in an OR83b-independent manner in different recombinant expression systems [12], [44], [50], [54], [55]. While further mechanistic models have to await investigations on the mode of receptor G-protein coupling in the olfactory system, it is tempting to speculate that *Drosophila* ORs couple to heterotrimeric G-proteins although having an inverse membrane topology compared to other GPCRs. Human adiponectin receptors (AdipoRs) and membrane progestin receptors (mPRs), belonging to the PAQR (Progestin, AdipoQ-Receptor) family of proteins, are seven transmembrane receptors of a novel type that, similarly to *Drosophila* OR83b, share little sequence homology with other GPCRs [56], [57]. Interestingly, AdipoRs have been shown to have intracellular N- and extracellular C-termini [56], [58], but location of the termini of mPR is yet to be confirmed. Nevertheless, the fish mPR has been shown to be a plasma membrane protein whose activation leads to inhibition of adenylyl cyclase in a pertussis toxin-sensitive manner, consistent with mPR being a novel type of GPCR [57].

### Dual signaling mode in *Drosophila* chemosensory systems

Also *Drosophila* gustatory receptors (GRs) lack sequence homology with mammalian taste receptors or other GPCRs, but are distantly related to the large family of *Drosophila* ORs [59]. Although not unambiguously shown until now, ligand-gated channel properties of *Drosophila* GRs were suggested by *in situ* patch clamp recordings showing that an ion channel is directly gated by sucrose in fleshfly taste neurons [60]. Nevertheless, different G-protein mutant flies showed significantly decreased responses to sugars in comparison to control flies, but not a complete suppression of the responses [27]–[29]. The fact that CO_2_ detection, mediated by a heterodimer consisting of GR21a and GR63a expressed in the fly antennae, requires Gqα and Gγ30a *in vivo* provides additional evidence for GR-mediated signal transduction via a metabotropic mechanism [30], [59].

### cAMP signaling in the insect olfactory system

Gα_s_-proteins typically activate adenylyl cyclases to produce cAMP. Expression of a light-activated adenylyl cyclase in the olfactory neurons enabled us to show that a cAMP increase results in increased firing rates, providing hints for the existence of cAMP dependent excitatory signaling pathway. Also the effects of overexpression of a cAMP-phosphodiesterase (PDE) in olfactory neurons was indicative of an excitatory role for cAMP in the *Drosophila* olfactory system [61]. Cyclic nucleotide-activated currents were also described in cultured olfactory receptor neurons of the moth *Manduca sexta* [62]. Endogenous circadian rhythms of cyclic nucleotides, generated by varying octopamine levels, contribute to the control of the intracellular Ca^2+^ concentrations in ORNs from *Manduca* [38], [63], and generate more sensitive pheromone responses during the night. Whether cAMP levels are modulated with the endogenous circadian rhythm in *Drosophila* is not known at the moment, but could be investigated in future studies. It is unclear if the cAMP rise in *Manduca* increases the spontaneous activity of ORNs as this is not yet examined in the moth, but the PAC mediated cAMP modulation that we describe resulted in modulation of the spike rate in *Drosophila* ORNs, and suggests that this might also take place in other insects as already supposed by Monika Stengl [49].

The heteromeric OR/OR83b complexes could mediate spontaneous activity by forming constitutively open non-specific cation channels [14], [48], and it was proposed that the insect receptor–ion channel complex functions in the control of membrane potential oscillations [48], [49]. Since the open probability of the OR/OR83b complexes was shown to be regulated by cAMP [48], cAMP may regulate the membrane potential of the ORNs via the OR/OR83b complex.

We found here, that cAMP also induces increased spike rates in ORNs from OR83b k.o. flies, showing that there are more cAMP targets in the cells. Cyclic nucleotide–gated channels and I_h_ channels as potential cAMP targets are known to be expressed in the third antennal segment of a *Drosophila* [64]–[67] and mutants of the *ether a go-go* (eag) gene encoding a cyclic nucleotide–modulated K^+^ channel show olfactory deficits [68]. Interestingly, in sensory neurons in the basal layer of the mouse VNO K^+^ channels encoded by *ether-à-go-go*-related gene (ERG) function as key determinants of cellular excitability, and an increase in ERG channel expression extends the dynamic range of the stimulus–response function in basal vomeronasal sensory neurons [69]. *Drosophila* olfactory neurons usually display background firing in the absence of odorants, cAMP dependent eag activation might provide an OR based mechanism of homeostatic plasticity in the periphery of the olfactory system, and could adjust the neurons to a desired output range.

### Further evidences for G-protein signaling in *Drosophila* olfaction

An interesting observation is, that regulation of odor responses in *Drosophila* antennae by cell-autonomous circadian clocks is mediated by the levels of the G-protein receptor kinase GPRK2 [70], which influences the distribution of ORs in the sensory cells [71]. Rhythmic changes in spike amplitude were observed in-phase with the rhythms of odor-evoked EAG responses, but spontaneous or odor-evoked spike frequency of olfactory neurons did not change [70]. GPRK2 therefore has to influence an OR dependent signaling pathway downstream of the OR ion channel activity, possibly involving heterotrimeric G-proteins. Interestingly, we also have initial observations that the subcellular distribution of Gαs changes upon odorant exposure, with Gαs showing a marked redistribution from the dendrites of the sensory neurons to the base of the sensilla upon odorant exposure, providing further indication of an involvement of the protein in the signaling cascade (Figure S6).

There is other evidence for involvement of G-protein signaling in the *Drosophila* olfactory system. The exposure to nutrient-derived odorants in Drosophila was shown to modulate life span in an *Or83b* dependent manner [72]. Longevity was extended when olfactory signaling was suppressed in *Or83b*-expressing neurons through the disruption of G-protein signaling [72], and it was speculated that G-protein function in an odor-induced signaling cascade in sensory neurons influences life span [72]. Moreover, accompanying microarray analysis revealed that aging and diet mostly affect expression of *Drosophila* odorant binding proteins and genes involved in regulating G-protein signaling.

Last but not least, there is more than single evidence that arrestins modulate olfactory signal transduction in insects [73]–[76], indicating that the receptors retained some properties of classical G-protein coupled receptors.

### Conclusions

Taken together, we provide here strong evidence for the involvement of Gαs in olfactory signal transduction in *Drosophila* and signaling of recombinant *Drosophila* ORs, although these proteins have been shown to have an unusual membrane topology and comprise a new class of ligand-activated non-selective cation channels [14], [48]. Whether G-protein activation in the ORNs is mediated via ORs with intracellular C-termini or via a G-protein-binding domain at the N-terminus remains to be examined in future studies. There are experimental evidences for and against G-protein signaling in *Drosophila* olfaction. Our data are consonant with a recently proposed dual-activation model [77], due to which the primary response is generated by activation of ligand-gated ion channels, followed by a G-protein-mediated potentiation or modulation of the ionotropic response. Insects seem to have not only one principal odor transduction cascade, but different cascades which may allow for adjustment of the odor response depending on the endogenous rhythms, behavioural states, and the properties of the odorants [49]. Although further mechanistic models have to await investigations on the mode of receptor G-protein coupling in the olfactory system, it is tempting to speculate that *Drosophila* ORs couple to heterotrimeric G-proteins although having an inverse membrane topology compared to other GPCRs. This duality in signaling might ensure the precision of the underlying transduction mechanisms and allow the insect to cover a wide dynamic range, highly important due to the behavioral relevance of olfactory signaling in insects.

## Methods

### *Drosophila* stocks

All fly stocks were maintained on conventional cornmeal-agar-molasse medium under a 12-hr-light:12-hr-dark cycle at 18°C or 25°C. The following fly stocks were kindly provided: OR83b-Gal4 (John Carlson, Yale University, USA), UAS-mCD8-GFP, Gr21a-Gal4, Or22a-Gal4 (Bloominton Stock Center), w[1118]; P{ry[+t7.2] = 70FLP}10 (Bloomingtom Stock Center #6938), UAS-Gαs-wt and UAS-GαsQ215L (Cahir O'Kane, University of Cambridge, England), UAS-Gαo-wt, UAS-GαoQ205L, UAS-Gαo-G203T, UAS<*[w* ^+^ *]*<Ctx and UAS<*[w* ^+^ *]*<Ptx (Andrew Tomlinson, Columbia University, USA); UAS-Gαq and UAS-GαqQ203L (Gaiti Hasan, Tata Institute of Fundamental Research, India); UAS-Gαi-wt, UAS-GαiQ205L and UAS-GαiRNAi (Juergen Knoblich, Institute of Molecular Biotechnology, Austria); UAS-Pacα (Martin Schwärzel, Freie Universität Berlin, Germany); *Or83b^2^/Or83b^2^* (*Or83b^-/-^)* (Leslie Vosshall, Rockefeller University, USA). For the generation of UAS-Gαs-GFP flies, the transgenic construct was injected into yw embryo by the VANEDIS *Drosophila* injection service (Oslo, Norway) using standard procedures.

### Construction of expression plasmids

The C-terminus of the human G-protein alpha subunit 16 (Gα_16_ΔC44) was amplified from an existing construct (pCISG16 [78]) by PCR using the following primers 5′-cgggatccatggcccgctcgctgacc-3′; 5′-cggatatcgccctcggggccgtccac-3′. The PCR fragment was cloned into the BamHI and EcoRV sites of the mammalian expression vector pCDNA3 (Invitrogen). Different *Drosophila* G-protein C-terminal fragments were generated from *Drosophila* head cDNA by polymerase chain reaction and fused into the EcoRV and XbaI sites of the pCDNA3-Gα_16_-ΔC44 construct. The primers used for the PCR were: Gαs-fw, 5′-gctagcggagacggaaaac-3′, Gαs-rw, 5′-cgtctagatagtaacaattcatattgacgaagg-3′; Gαq-fw, 5′-cattagtttagatacatataa-3′, Gαq-rw, 5′-cgtctagatagaaacagattactttcttttagg-3′; Gαo-fw, 5′-aacaaatcaacctcaaaag-3′, Gαo-rw, 5′-cgtctagataggtacagtccacagccgcg-3′; Gαi-fw, 5′-aacaagcgaaaagaccaaaagg-3′, Gαi-rw, 5′-cgtctagataggaataagccaatttgtttcag-3′; Gα73B-fw, 5′-ctgggtacctcggaaagggag-3′, Gα73B-rw, 5′-cgtctagataggaataggcccatgctggacac-3′.

### Cell culture and transfection

Experiments using heterologous expression system were performed in HEK293 cells, which were maintained under standard conditions in MEM supplemented with 10% FBS, 100 units/ml penicillin and streptomycin, and 2 mM L-glutamine. Transfections were done using a standard calcium phosphate precipitation technique.

### Single cell Ca^2+^ imaging

For calcium imaging experiments, HEK293 cells were transfected with OR43a, OR83b and the respective G-protein chimera or full length human Gα_16_ as control (ratio 1∶2∶4). Two days after transfection, culture medium was removed and replaced with Ringer solution. Cells were incubated (45 min) in Ringer solution containing 7.5 µM Fura-2-AM (Invitrogen) at room temperature. Ratiofluometric Ca^2+^ imaging was performed as described [51], [79] using a Zeiss inverted microscope equipped for ratiometric imaging with a xenon arc lamp, a multiway wavelength illumination system POLYCHROME II for excitation, a cooled charge-coupled device (CCD) camera, and WinNT based T.I.L.L.-Vision software to collect and quantitfy spatiotemporal Ca^2+-^dependent fluorescence signals (* f*340/*f*380 ratio). Cells were exposed to the odorant cyclohexanone (500 µM) for 10 s using a specialized microcapillary application system that transiently superfuses all cells in the field of view from one of six user-selected capillary tubes, tube tips were in close proximity to the optical field. Ringer's solution continuously superfused all the cells in the dish between application of test solutions. Images were acquired from up to 15 cells in a randomly selected field of view, and integrated fluorescence ratios (* f*340/*f*380 ratio) were measured.

### [^35^S]GTPγS binding assay

Transfected cells were homogenized in 10 mM HEPES and 10 mM EDTA (pH 7.4) and membrane preparations were prepared. [^35^S]GTPγS binding was assayed in a final volume of 100 µl (pH 7.4), containing 10 mM HEPES, 100 mM NaCl, 10 mM MgCl_2_, 100 mM guanosine 5′-diphosphate (GDP) and 0.1 nM [^35^S]GTPγS. The incubation was started by the addition of membrane suspension (about 50 µg of membrane protein per reaction) and was carried out in triplicate for 30 min at 30°C. The reaction was terminated by addition of 3 ml of ice-cold filter wash buffer and rapidly vacuum filtered through glass fiber filters (Whatman GF/C). Filters were washed three times with 3 ml of wash buffer, transferred to scintillation vials, and counted in a Packard Tri-Carb liquid scintillation counter.

### Western blotting and RT-PCR

The third antennal segments were collected from 100 flies (3–5 days old). RNA was isolated with Trizol Reagent (Invitrogen) and cDNA was synthesized using MMLV reverse transcriptase (Invitrogen) and oligo (dT12-18) primer. Amplifications were performed with 1 ng cDNA and specific primer pairs for Gα_s_ (Gαs440-fw: 5′-TTCTTCAAACCTATGAGAGG-3′ and Gαs661-rw: 5′-TCCTACGCTCGTCCCGCTGG-3′).

For western blotting, 200 antennae were homogenized in Laemmli buffer (30% glycerol, 3% SDS, 125 mM Tris/Cl, pH 6.8), resolved by 10% SDS-PAGE and transferred to nitrocellulose membrane (Protran; Schleicher & Schuell). Transfected HEK293 cells were harvested two days after transfection. Cells were homogenized and post-nuclear supernatants were mixed with Laemmli buffer, seperated on 10% SDS-PAGE gels and blotted. The membranes were incubated with primary anti-Gα_olf_ (Santa Cruz), with and without pre-incubation with a specific blocking peptide (Santa Cruz), or anti-Gα16 (sc-7416, Santa Cruz) antibodies and detection was performed with ECL plus (Amersham) on Hyperfilm ECL (Amersham).

### Immunohistochemistry

Fly heads were cut, fixed for 3 hr in 4% paraformaldehyde at 4°C and subsequently incubated overnight in 25% sucrose in *Drosophila* Ringer's solution (182 mM KCl, 46 mM NaCl, 3 mM CaCl_2_, 10 mM Tris-HCl, pH 7.2). Cryosectioning was performed to produce 12 µm sections. After blocking with 5% goat serum in Ringer's, a polyclonal anti-Gα_olf_ antibody (Santa Cruz), a polyclonal anti-OR83b antibody (University of Texas-Southwestern Medical Center [80]), or anti-GFP antibody (Abcam) were applied to the sections overnight at 4°C. After washing, the secondary anti goat antibody coupled to A546 (Invitrogen) was applied for 1 hr at room temperature. Pictures were taken with a Zeiss confocal microscope (LSM510 Meta; Zeiss).

### Electrophysiology

The electroantennogram (EAG) responses of wt and G-protein mutant flies were recored as previously described [12], [81]. Briefly, 2–3-day-old flies were mounted in truncated micropipette tips with the anterior portion of the head protruding from the end of the tip. The indifferent electrode was inserted into the haemolymph of the head capsule. The recording electrode was placed on the frontal surface of the anterior aspect of the antenna. After obtaining a stable baseline, EAG recordings were initiated by a short pulse of odor (0.3 s), applied into an air stream that was directed toward the antenna. Duration of the pulse was electronically controlled. Total airflow was 2.8 l/min (tube diameter  =  7 mm). The fraction of the stimulus or the respective blank controls was 0.4 ml/min, which was injected into a background stream of 2.4 l/min. When no stimulus was applied, a respective compensatory airstream was injected to keep the input air pressure on the fly constant. 50 µl of the odorant solutions were pipetted on filter paper strips that were inserted in 5 ml plastic serological syringes. The volume of the pipettes was adjusted to 4 ml, through which the stimulus airstream was directed during the application. Since the stimulus airstream was injected into the background stream before application to the antenna, the odorant containing gas phase was diluted to 14% prior application. The final odorant concentration in the gas phase that reaches the antenna can not be determined precisely, only different dilutions of the odorant can therefore be compared. CO_2_ was applied in a similar manner resulting in a final concentration of 14% due to the dilution with the background air stream. All odorants were purchased from Sigma in the highest available purity and were dissolved in paraffin oil at the given concentrations. Odorants used were cyclohexanol, benzaldehyde, heptanone and ethyl acetate. Inter-stimulus intervals were 30 s to avoid adaptation of the olfactory system to the stimuli.

The procedures of extracellular single-unit recording was essentially similar as previous report [82]. Canton-S flies aged < 1 week were used for wt recordings. *hs-flp; Or83b-Gal4, UAS<[w^+^]<CTX* flies were recorded after standard heat shock (one hour, 38°C) at age 4-5 days. Electrical activity of the neurons was recorded extracellularly by placing an electrode filled with sensillum lymph ringer in the base of the sensillum. A reference electrode filled with the same ringer was placed in the eye. Signals was amplified using a patch-clamp amplifier (L/M-PC, List-Medical Electronic, Darmstadt, Germany) in voltage-clamp mode and fed into a computer via a 12-bit analog/digital converter (Digidata 1200A; Molecular Devices, CA, USA). Impulses during the 1 s period before stimulation and the 1 s during stimulation were counted off-line using the Mini Analysis software (Synaptosoft, Decatur, GA, USA). The spikes were sorted into different groups according to their amplitude as previously described [83]. Each sensillum was tested with multiple odorants, and no more than two sensilla were analyzed per fly. Blue light exposure was accomplished with fluorescence illumination channel of the microscope using appropriate filters.

## Supporting Information

Figure S1 **RT-PCR analysis of Gα subunit expression in** ***Drosophila*** **antenna.** (**A**) RT-PCR results revealed expression of all Gα subunits transcripts in the antenna, with a higher expression level of Gαq1, Gαq3, Gαs, CG17766 and CG17760 as compared to that of Gαf, CG3004 and CG30054. Gαq3 and the retina specific Gαq1 are transcribed from *Gα49B* (CG17759) (Talluri et al., 1995). The different lanes correspond to: 1:Marker; 2:Gαf; 3:Gαq1eye-1; 4:Gαq3-1; 5:Gαq1eye-2; 6:Gαq3-2; 7:Gαs; 8:CG30054, 9:CG17766; 10: CG17760; 11:CG3004 (primer sequences are provided in Text S1). (**B**) Heterotrimeric G-protein α subunits are divided into 4 classes based on their sequence similarity and downstream effectors. Together with some well-identified Gα subunits from other species, a phylogenetic tree of Gα subunits was generated by the MegAlign program using the Clustal V method. As shown, 3 classes of vertebrate Gα proteins can also be identified from *Drosophila*. The ungrouped G-proteins are Gαf, CG3004 and CG17766, which show low similarities with the classified Gα subunits. Phylogenetic tree of Gα subunits from *Drosophila melanogaster, Mus musculus, Homo sapiens, Anopheles gambiae, Panulirus argus*, and *Rattus norvergicus*.
(TIF)

Figure S2 **EAG recordings of flies with impaired G-protein signaling.** (**A**) EAG amplitudes (in mV) of Gα_q_ mutant flies expressing constitutively active (GTPase deficient) Gα_q_ (n>10 flies were recorded), expression of the UAS construct was driven by *Or83b-Gal4*. Odorants used were ethyl actetate, cyclohexanol and benzaldehyde, each in concentrations of 10^-3^, 10^-2^, 10^-1^, and undiluted. The differences between Gαq flies and wt flies were statistically checked (pairwise) by unpaired Student's *t* tests; significance levels were set according to the Bonferroni post hoc test for *k* = 4 means per odorant, *P≤0.0125, **P≤0.0025, p values are given on top the respective bars. (**B**) EAG amplitudes (in mV) of wild-type flies (control), wild-type flies where standard ringer solution was microinjected in the third antennal segment (ringers), and flies where CTX (diluted in ringer solution, 100 ng/ml) was injected in the third antennal segment (CTX). Error bars represent s.e.m.
(TIF)

Figure S3 **Higher magnification of single sensillum recording from ab1 sensillum in wt flies.** Spikes corresponding to activation of the different neurons (ab1A-ab1D) are labeled.
(TIF)

Figure S4 **Expression of constitutively active Gα_s_.** (**A**) Shown are traces from the ab1 sensillum of wt and *Or83b-Gal4; UAS-Gα_s_-GTP* flies during the 1000 ms ethyl acetate application period. (**B**) Summary of the responses in single unit recordings of ab1A neurons in *Or83b-Gal4; UAS-Gα_s_-GTP* flies, expressing a GTPase deficient Gα_s_ mutant. Specifically the initial increase in spike rates upon application of ethyl acetate (1∶100) was analyzed, number of spikes in 50 ms is counted and shown here. Differences between data points were statistically checked by the unpaired Student's *t* test, p values are indicated on top of each bar. Significance levels were set according to the Bonferroni post hoc test for *k* = 20 means, P≤0.0025; the intial response in the first 50 ms was significantly different between both flies. Error bars represent s.e.m.
(TIF)

Figure S5 **Control single sensillum recordings with PAC flies.** Shown are traces from the ab1 sensillum from Or83b-/- flies; light exposed Or83b-/- flies showing that the sensillum does not respond to light exposure; PAC expressing OR83b-/- flies showing no increase in spike rate due to PAC expression; PAC expressing OR83b-/- flies exposed to CO_2_, showing that CO_2_ responses are normal; PAC expressing OR83b-/- flies exposed to odorants, showing that PAC expression does not restore any odorant induced activity in the neurons; PAC expressing OR83b-/- flies exposed to light, showing increase in spike rate due to PAC mediated cAMP increase.
(TIF)

Figure S6 **G-protein redistribution upon odorant exposure.** (**A**) Higher magnification pictures of Gα_s_ staining (red), showing that Gα_s_ is localized in the dendrites of the sensory neurons in odorant deprived animals, but is translocated to the cell body and the basis of the sensilla upon odorant exposure. (**B**) To confirm the observed redistribution of activated Gα_s_, a fly line was generated expressing a Gα_s_-GFP fusion protein under control of the UAS promoter (*OR83b-Gal4; UAS- Gα_s_-GFP*), a schematic drawing of the fusion construct is provided. Fusion of GFP to either C- or N-terminus is critical for G-protein α subunits, since the NH_2_ region is important for association with the G_βγ_ subunits and the COOH terminal is required for interaction with receptor, but functional Gα-GFP fusion proteins were obtained by inserting GFP into an internal loop. Expression of a similar fusion protein in olfactory neurons (*Gal4-OR83b;UAS-Gα_s_-GFP*) resulted in green fluorescence in the sensory neurons, and was identified in the third antennal segment by immunoblotting. In odorant deprived animals, the fusion protein was localized in the cell bodies and in the sensilla, but in odorant exposed flies Gα_s_-GFP could no longer be detected in the dendrites of the sensory neurons, indicating displacement of the fusion protein from the plasma membrane throughout the cytoplasm of the cells.
(TIF)

Table S1 **G-protein mutant strains used in this study.**
(DOCX)

Table S2 **EAG measurements on mutant flies (Results of** **Figure 1C** **).**
(DOCX)

Text S1 **Supplementary methods.** RT-PCR analysis of Gα subunits in *Drosophila* antenna.
(DOCX)
