# Supplementary figures and images for: The Stimulatory Gαs Protein Is Involved in Olfactory Signal Transduction in Drosophila
Source: PLoS One. 2011 Apr 7;6(4):e18605. doi: 10.1371/journal.pone.0018605 (PMC3072409; doi:10.1371/journal.pone.0018605)

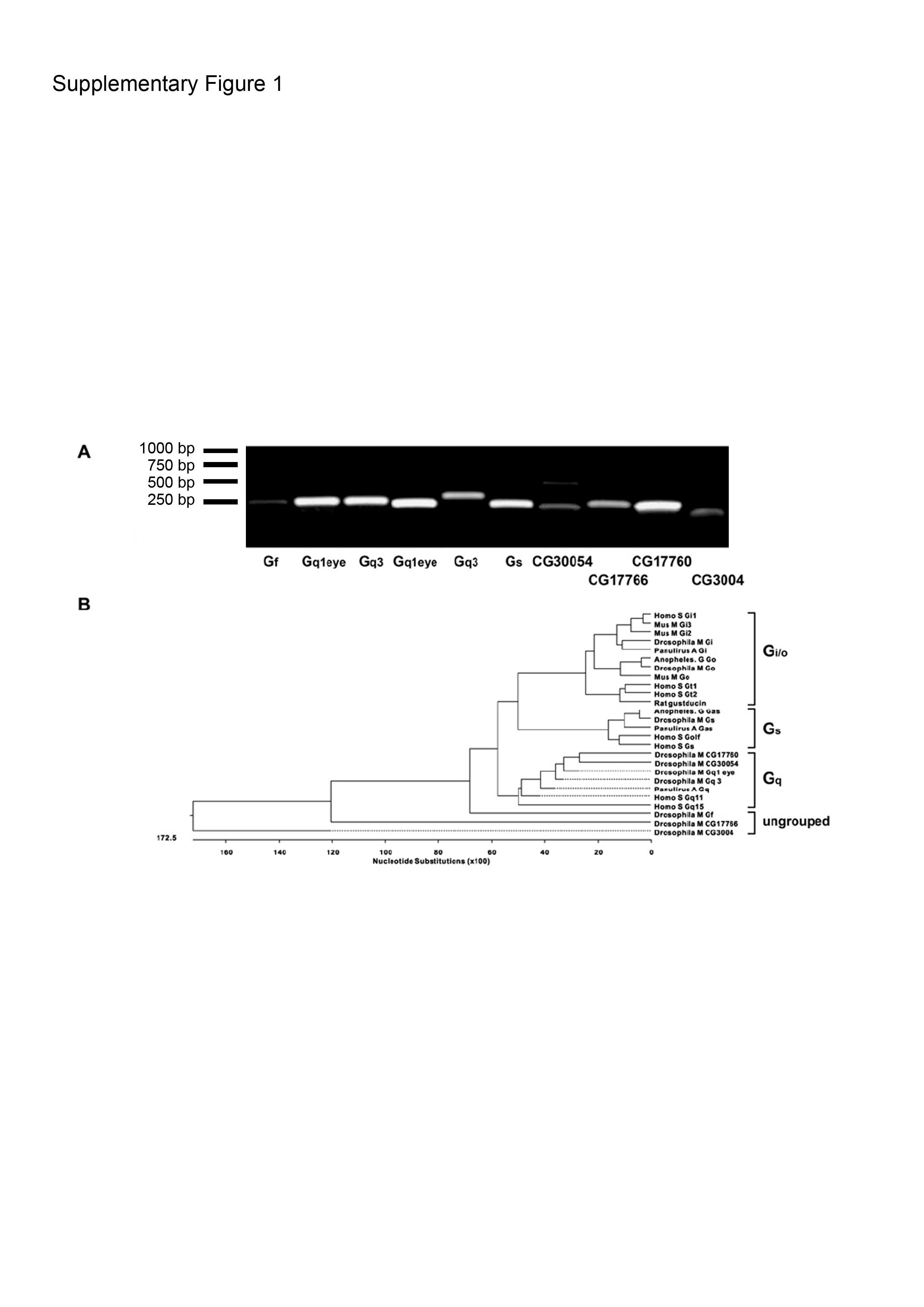

Supplement: Figure S1 — RT-PCR analysis of Gα subunit expression in Drosophila antenna. (A) RT-PCR results revealed expression of all Gα subunits transcripts in the antenna, with a higher expression level of Gαq1, Gαq3, Gαs, CG17766 and CG17760 as compared to that of Gαf, CG3004 and CG30054. Gαq3 and the retina specific Gαq1 are transcribed from Gα49B (CG17759) (Talluri et al., 1995). The different lanes correspond to: 1:Marker; 2:Gαf; 3:Gαq1eye-1; 4:Gαq3-1; 5:Gαq1eye-2; 6:Gαq3-2; 7:Gαs; 8:CG30054, 9:CG17766; 10: CG17760; 11:CG3004 (primer sequences are provided in Text S1). (B) Heterotrimeric G-protein α subunits are divided into 4 classes based on their sequence similarity and downstream effectors. Together with some well-identified Gα subunits from other species, a phylogenetic tree of Gα subunits was generated by the MegAlign program using the Clustal V method. As shown, 3 classes of vertebrate Gα proteins can also be identified from Drosophila. The ungrouped G-proteins are Gαf, CG3004 and CG17766, which show low similarities with the classified Gα subunits. Phylogenetic tree of Gα subunits from Drosophila melanogaster, Mus musculus, Homo sapiens, Anopheles gambiae, Panulirus argus, and Rattus norvergicus. (TIF) [file pone.0018605.s001.tif]

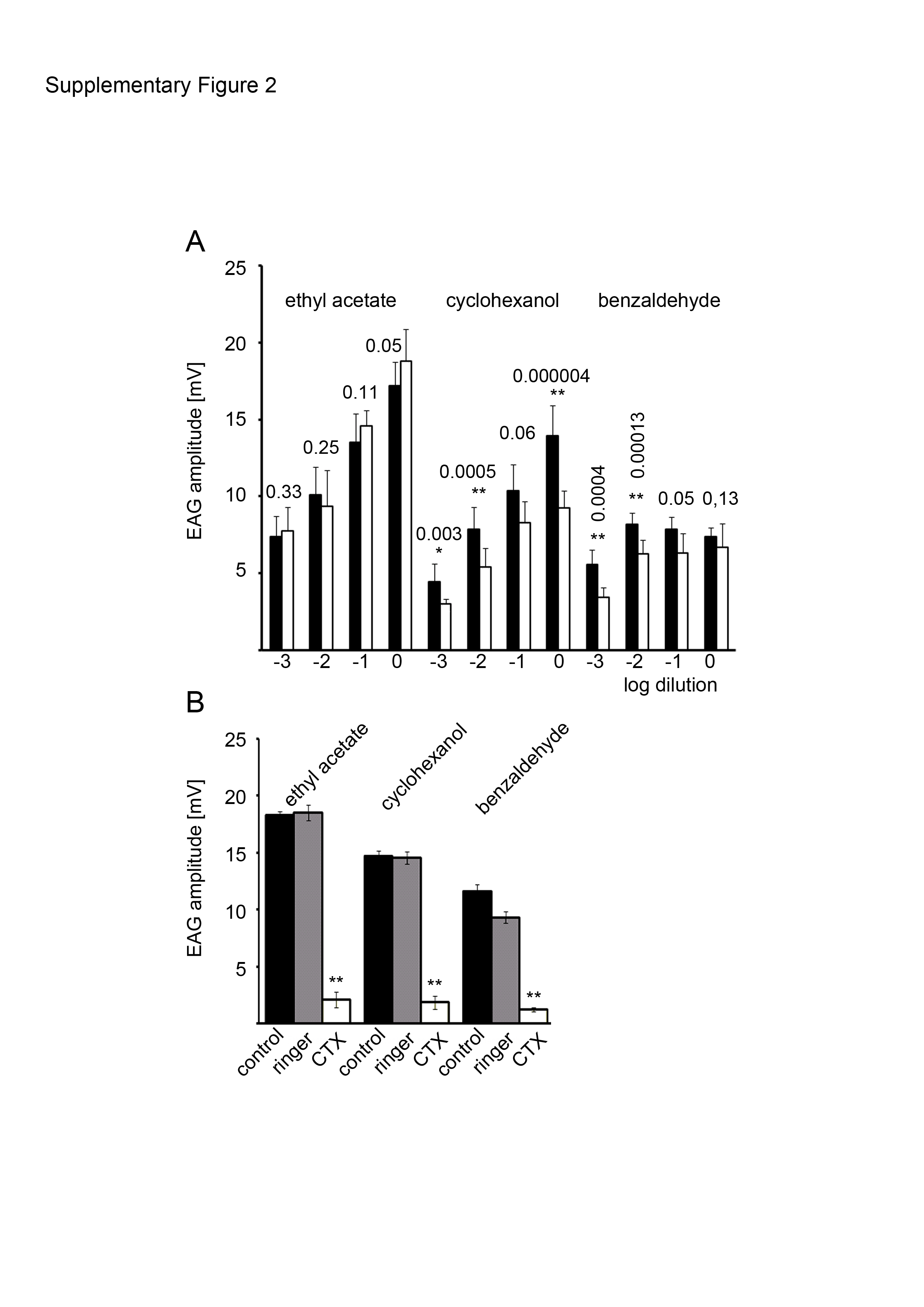

Supplement: Figure S2 — EAG recordings of flies with impaired G-protein signaling. (A) EAG amplitudes (in mV) of Gαq mutant flies expressing constitutively active (GTPase deficient) Gαq (n>10 flies were recorded), expression of the UAS construct was driven by Or83b-Gal4. Odorants used were ethyl actetate, cyclohexanol and benzaldehyde, each in concentrations of 10-3, 10-2, 10-1, and undiluted. The differences between Gαq flies and wt flies were statistically checked (pairwise) by unpaired Student's t tests; significance levels were set according to the Bonferroni post hoc test for k = 4 means per odorant, *P≤0.0125, **P≤0.0025, p values are given on top the respective bars. (B) EAG amplitudes (in mV) of wild-type flies (control), wild-type flies where standard ringer solution was microinjected in the third antennal segment (ringers), and flies where CTX (diluted in ringer solution, 100 ng/ml) was injected in the third antennal segment (CTX). Error bars represent s.e.m. (TIF) [file pone.0018605.s002.tif]

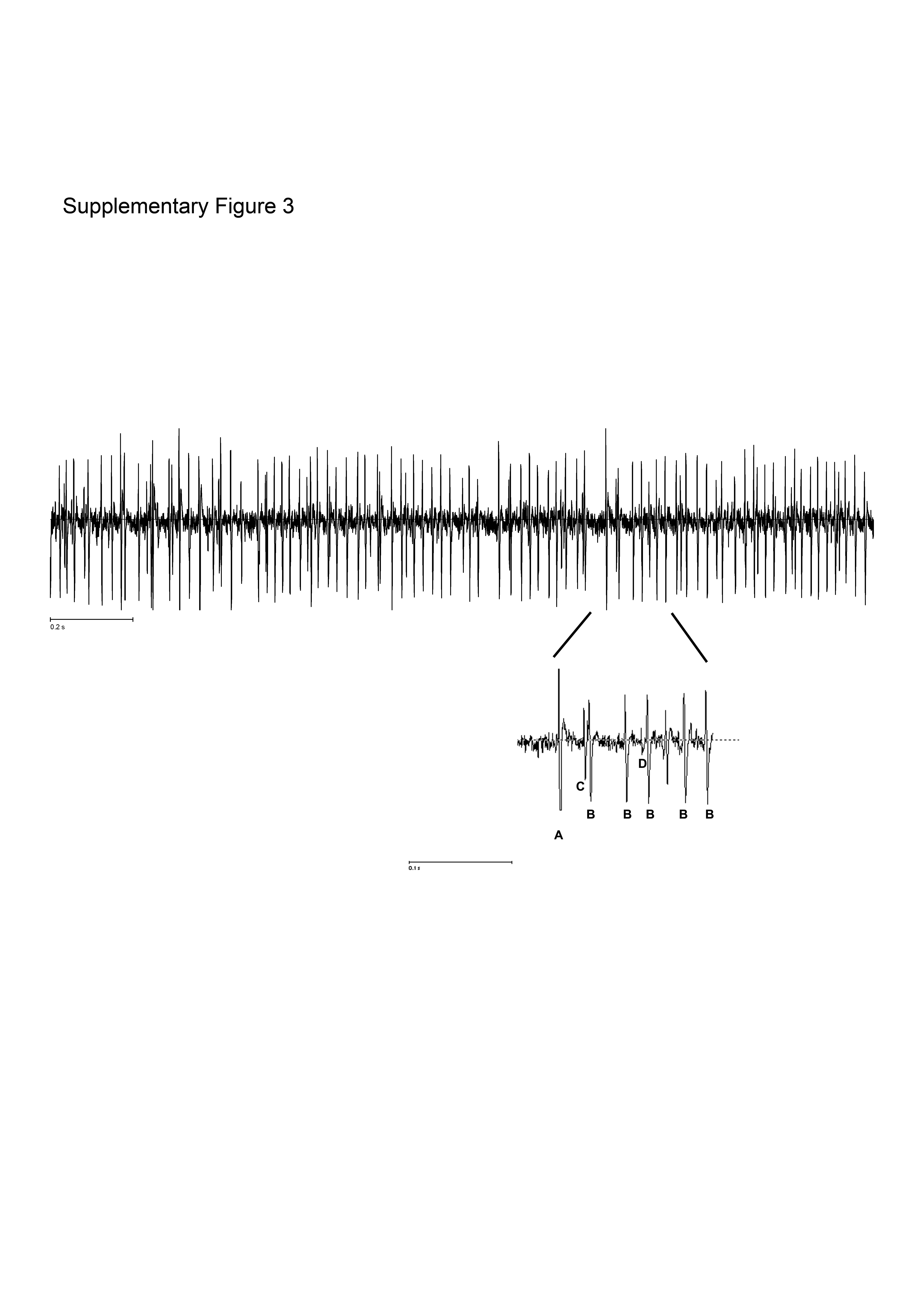

Supplement: Figure S3 — Higher magnification of single sensillum recording from ab1 sensillum in wt flies. Spikes corresponding to activation of the different neurons (ab1A-ab1D) are labeled. (TIF) [file pone.0018605.s003.tif]

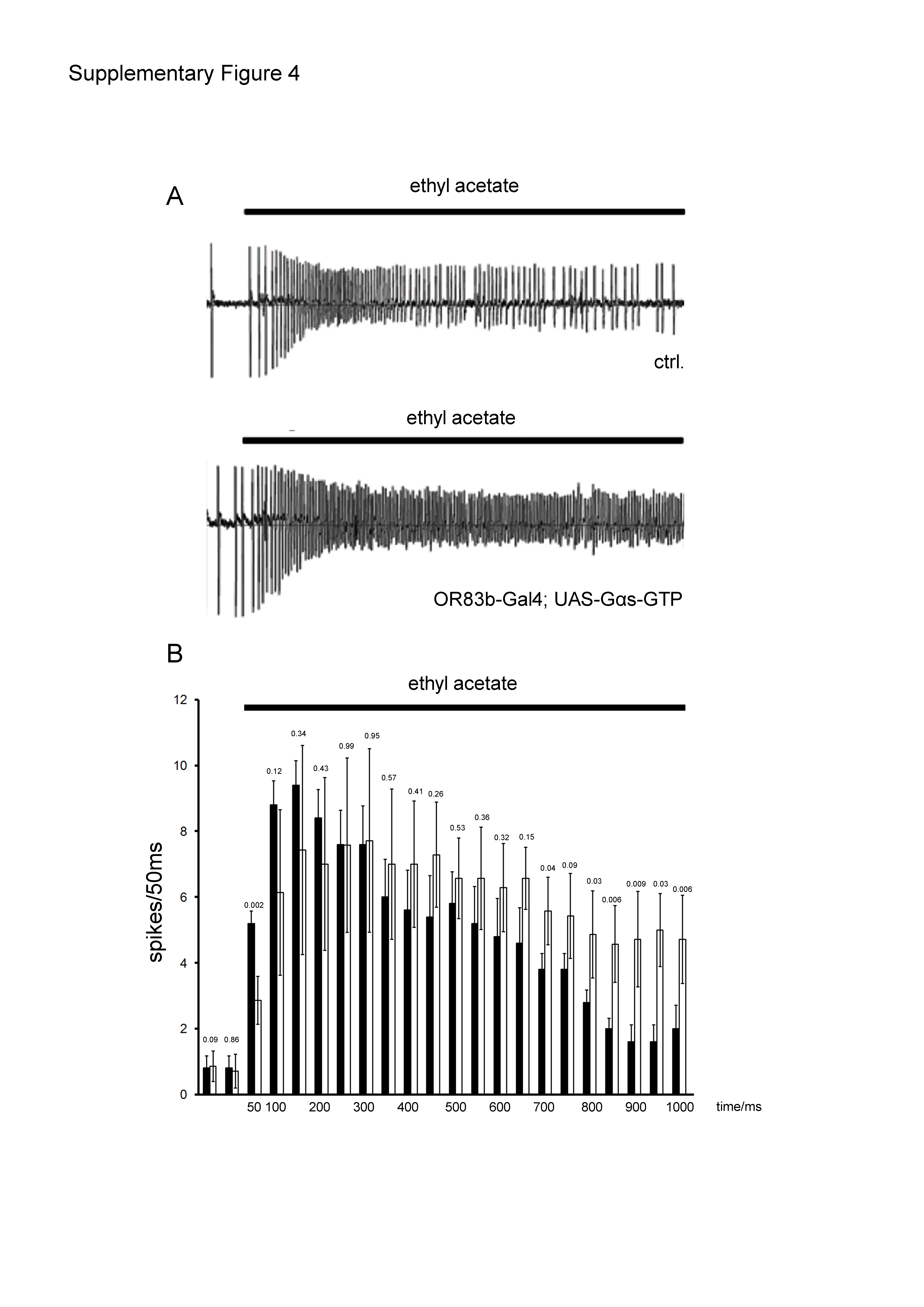

Supplement: Figure S4 — Expression of constitutively active Gαs. (A) Shown are traces from the ab1 sensillum of wt and Or83b-Gal4; UAS-Gαs-GTP flies during the 1000 ms ethyl acetate application period. (B) Summary of the responses in single unit recordings of ab1A neurons in Or83b-Gal4; UAS-Gαs-GTP flies, expressing a GTPase deficient Gαs mutant. Specifically the initial increase in spike rates upon application of ethyl acetate (1∶100) was analyzed, number of spikes in 50 ms is counted and shown here. Differences between data points were statistically checked by the unpaired Student's t test, p values are indicated on top of each bar. Significance levels were set according to the Bonferroni post hoc test for k = 20 means, P≤0.0025; the intial response in the first 50 ms was significantly different between both flies. Error bars represent s.e.m. (TIF) [file pone.0018605.s004.tif]

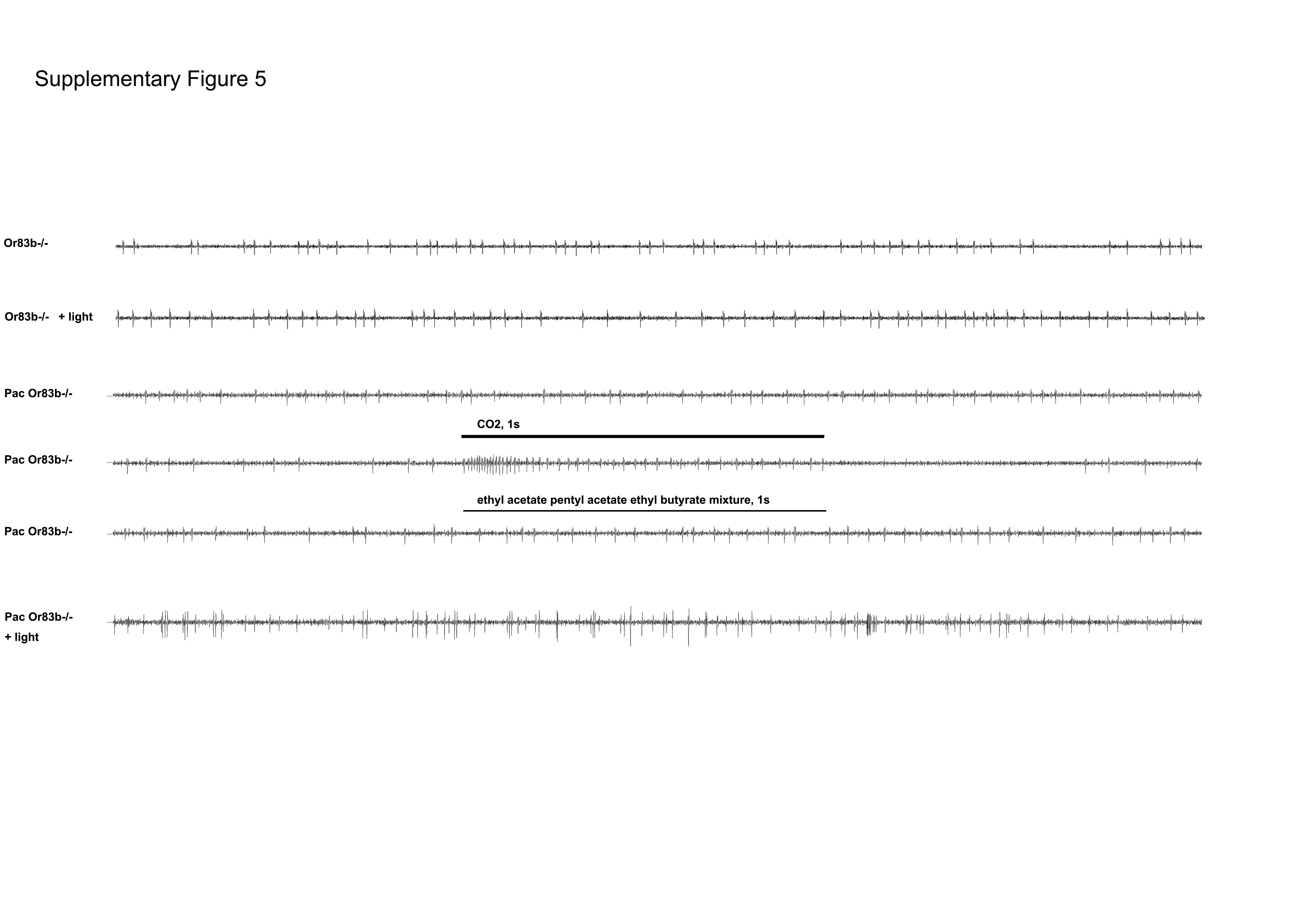

Supplement: Figure S5 — Control single sensillum recordings with PAC flies. Shown are traces from the ab1 sensillum from Or83b-/- flies; light exposed Or83b-/- flies showing that the sensillum does not respond to light exposure; PAC expressing OR83b-/- flies showing no increase in spike rate due to PAC expression; PAC expressing OR83b-/- flies exposed to CO2, showing that CO2 responses are normal; PAC expressing OR83b-/- flies exposed to odorants, showing that PAC expression does not restore any odorant induced activity in the neurons; PAC expressing OR83b-/- flies exposed to light, showing increase in spike rate due to PAC mediated cAMP increase. (TIF) [file pone.0018605.s005.tif]

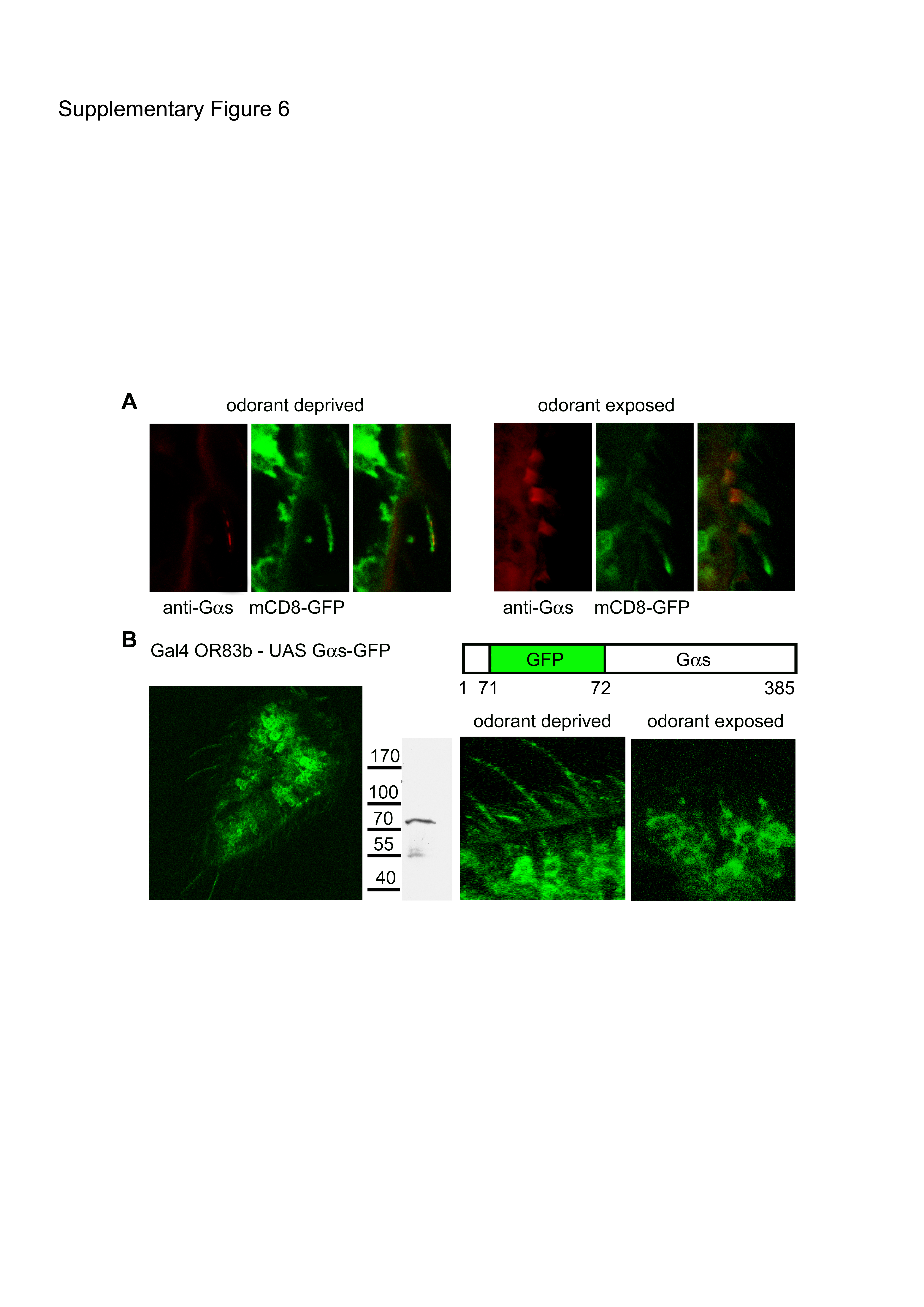

Supplement: Figure S6 — G-protein redistribution upon odorant exposure. (A) Higher magnification pictures of Gαs staining (red), showing that Gαs is localized in the dendrites of the sensory neurons in odorant deprived animals, but is translocated to the cell body and the basis of the sensilla upon odorant exposure. (B) To confirm the observed redistribution of activated Gαs, a fly line was generated expressing a Gαs-GFP fusion protein under control of the UAS promoter (OR83b-Gal4; UAS- Gαs-GFP), a schematic drawing of the fusion construct is provided. Fusion of GFP to either C- or N-terminus is critical for G-protein α subunits, since the NH2 region is important for association with the Gβγ subunits and the COOH terminal is required for interaction with receptor, but functional Gα-GFP fusion proteins were obtained by inserting GFP into an internal loop. Expression of a similar fusion protein in olfactory neurons (Gal4-OR83b;UAS-Gαs-GFP) resulted in green fluorescence in the sensory neurons, and was identified in the third antennal segment by immunoblotting. In odorant deprived animals, the fusion protein was localized in the cell bodies and in the sensilla, but in odorant exposed flies Gαs-GFP could no longer be detected in the dendrites of the sensory neurons, indicating displacement of the fusion protein from the plasma membrane throughout the cytoplasm of the cells. (TIF) [file pone.0018605.s006.tif]
