# Supplementary material for: The Stimulatory Gαs Protein Is Involved in Olfactory Signal Transduction in Drosophila
Source: PLoS One. 2011 Apr 7;6(4):e18605. doi: 10.1371/journal.pone.0018605 (PMC3072409; doi:10.1371/journal.pone.0018605)
Supplement: Table S1 — G-protein mutant strains used in this study. (DOCX) [file pone.0018605.s007.docx]

**Supplementary Table 1**. **G-protein mutant strains used in this study**

**Abbr. Full description Strain properties**

wt Canton S, wild type

Gαo *OR83b-Gal4; UAS-Gαo* overexpression of wt Gαo in OR83b neurons

Gαo-GTP *OR83b-Gal4; UAS-Gαo-GTP* overexpression of GTP bound Gαo, constitutive

active G-protein variant (deficient GTPase activity)

Gαo-GDP *OR83b-Gal4; UAS-Gαo-GDP* overexpression of GDP bound Gαo, inactive G-

protein variant

PTX Pertussis toxin inhibits specifically Goα by catalyzing ADP-

ribosylation of Gαo [31,32]

Gαq-GTP *OR83b-Gal4; UAS-Gαq-GTP* overexpression of GTP bound Gαq, constitutive

active G-protein variant (deficient GTPase activity)

Gαi-wt *OR83b-Gal4; UAS-Gαi* overexpression of wt Gαi in OR83b neurons

Gαi-GTP *OR83b-Gal4; UAS-Gαi-GTP* overexpression of GTP bound Gαi, constitutive

active G-protein variant (deficient GTPase activity)

Gαs-wt *OR83b-Gal4; UAS-Gαs* overexpression of wt Gαs in OR83b neurons

Gαs-GTP *OR83b-Gal4; UAS-Gαs-GTP* overexpression of GTP bound Gαs, constitutive

active G-protein variant (deficient GTPase activity)

CTX Cholera toxin ADP-ribosyltransferase activating Gα_s_ [33]

OR83b K.O. *Or83b^2^/Or83b^2^* Or83b knock-out flies [10]
