# Supplementary material for: The Stimulatory Gαs Protein Is Involved in Olfactory Signal Transduction in Drosophila
Source: PLoS One. 2011 Apr 7;6(4):e18605. doi: 10.1371/journal.pone.0018605 (PMC3072409; doi:10.1371/journal.pone.0018605)
Supplement: Table S2 — EAG measurements on mutant flies (Results of Figure 1C ). (DOCX) [file pone.0018605.s008.docx]

**Supplementary Table 2**. **EAG measurements on mutant flies (Results of Figure 1C)**

| Benzaldehyde (log dilution) | -4 | -3 | -2 | -1 |
| --- | --- | --- | --- | --- |
| Amplitude wt (mV) | 8,88 | 9,44 | 9,92 | 7,28 |
| s.e.m. | 0,326 | 0,435 | 0,258 | 0,372 |
| Amplitude CTX w/o h.s. (mV) | 8,88 | 9,28 | 8,92 | 6,56 |
| s.e.m. | 0,332 | 0,185 | 0,689 | 0,624 |
| Amplitude CTX w h.s. (mV) | 2,28 | 1,92 | 1,44 | 0,88 |
| s.e.m. | 0,472 | 0,445 | 0,24 | 0,258 |
| p-value to wt | 7,43E-06 | 2,06E-06 | 1,01E-08 | 1,80E-06 |
| p-value to CTX w/o h.s. | 7,17e-06 | 1,30E-05 | 1,59E-4 | 2,85E-04 |

| Ethyl acetate (log dilution) | -4 | -3 | -2 | -1 |
| --- | --- | --- | --- | --- |
| Amplitude wt (mV) | 18,44 | 16,08 | 12,08 | 8,88 |
| s.e.m. | 0,747 | 1,300 | 0,586 | 0,816 |
| Amplitude CTX w/o h.s. (mV) | 19,28 | 12,08 | 8,92 | 8,14 |
| s.e.m. | 1,237 | 1,328 | 1,563 | 0,626 |
| Amplitude CTX w h.s. (mV) | 3,56 | 2,28 | 1,52 | 0,96 |
| s.e.m. | 0,605 | 0,273 | 0,162 | 0,16 |
| p-value to wt | 4,63E-07 | 2,38E-05 | 1,65E-05 | 4,00E-04 |
| p-value to CTX w/o h.s. | 3,42E-05 | 3,66E-04 | 2,36E-3 | 1,88E-04 |

| Cyclohexanol (log dilution) | -4 | -3 | -2 | -1 |
| --- | --- | --- | --- | --- |
| Amplitude wt (mV) | 15,20 | 13,04 | 7,64 | 4,76 |
| s.e.m. | 0,469 | 0,483 | 0,293 | 0,075 |
| Amplitude CTX w/o h.s. (mV) | 15,76 | 12,72 | 8,60 | 5,32 |
| s.e.m. | 1,014 | 1,422 | 0,846 | 0,662 |
| Amplitude CTX w h.s. (mV) | 3,56 | 2,28 | 1,52 | 0,96 |
| s.e.m. | 0,133 | 0,102 | 0,098 | 0,133 |
| p-value to wt | 2,73E-06 | 1,11E-05 | 9,57E-06 | 4,43E-07 |
| p-value to CTX w/o h.s. | 1,42E-04 | 1,54E-04 | 1,22E-3 | 4,05-03 |
