# Supplementary material for: The Stimulatory Gαs Protein Is Involved in Olfactory Signal Transduction in Drosophila
Source: PLoS One. 2011 Apr 7;6(4):e18605. doi: 10.1371/journal.pone.0018605 (PMC3072409; doi:10.1371/journal.pone.0018605)
Supplement: Text S1 — Supplementary methods. RT-PCR analysis of Gα subunits in Drosophila antenna. (DOCX) [file pone.0018605.s009.docx]

**Supplementary methods**

**RT-PCR analysis of Gα subunits in *Drosophila* antenna**

We analyzed the expression of known transcript variants translated from genes that were classified to encode for heterotrimeric G-protein alpha subunits by RT-PCR. About 100 antennae were cut manually for RNA isolation.

The following primer pairs have been used for this analysis by RT-PCR (Suppl. Fig. 1):

Lane 11: CG3004f47: 5’-ACA CCA TTA AGG TGT GGC AGG-3’

CG3004r203: 5’-TTG GAC TCC AGG TCG TAC AGC-3’

Lane 10: CG17760f406: 5’-AAG GAG TGC TAC AAT CGT CG-3’

CG17760r612: 5’-TCG CTG ACC AGC GAC GTC CAC C-3’

Lane 9: CG17766f392: 5’-TCG AAC TCG GAT TCG GAA TTG-3’

CG17766r611: 5’-TGG TGC GCT TTA CCC AAT CTG-3’

Lane 8: CG30054f833: 5’-TGT ATT CGC ATT TGG TAG AC-3’

CG30054r1019: 5’-TCC TTA ACT GCA GCG AAC AC-3’

Lane 7: Gsfa440: 5’-TTC TTC AAA CCT ATG AGA GG-3’

Gsra661: 5’-TCC TAC GCT CGT CCC GCT GG-3’

Lane 6: G49bQ3af471: 5’-TCT CGA TCG TGT GGC TCA ACC-3’

G49bQ3Ar768: 5’-AGG GTA TGT AAT TAT AGT ACG-3’

Lane 5: G49bretinalqaf805: 5’-AAG AAG GAC TTG TTG GAA GAG-

G49bretinalqar1028: 5’-ATA ATT GTA TCT TTG ACA GC-3’

Lane 4: G49BQ3bf534: 5’-GCC CAC AAC AGG GAT AAT TG-3’

G49BQ3br786: 5’-TGA CGA ATT TTG AAA CCA AGG-3’

Lane 3: G49bretinalqbf766: 5’-CCT TGG TTT CAA AAT TCG TCA-

G49bretinalqbr1019: 5’-TCT TTG ACA GCG CAG AAC AC-

Lane 2: Galpha73Bf323: 5’-ATT TCG GCA GCT GTA CCA GCG-3’

Galpha73Br567: 5’-GTG CAG AAT GTC CTC GGT GC-3’
